# Evolution of Plastic Transmission Strategies in Avian Malaria

**DOI:** 10.1371/journal.ppat.1004308

**Published:** 2014-09-11

**Authors:** Stéphane Cornet, Antoine Nicot, Ana Rivero, Sylvain Gandon

**Affiliations:** 1 Centre d'Ecologie Fonctionnelle et Evolutive (CEFE), UMR CNRS 5175 - Université de Montpellier - Université Paul-Valéry Montpellier - EPHE, Montpellier, France; 2 Maladies Infectieuses et Vecteurs: Ecologie, Génétique, Evolution et Contrôle (MIVEGEC), UMR CNRS 5290-IRD 224-UM1-UM2, Montpellier, France; Institut Pasteur, France

## Abstract

Malaria parasites have been shown to adjust their life history traits to changing environmental conditions. Parasite relapses and recrudescences—marked increases in blood parasite numbers following a period when the parasite was either absent or present at very low levels in the blood, respectively—are expected to be part of such adaptive plastic strategies. Here, we first present a theoretical model that analyses the evolution of transmission strategies in fluctuating seasonal environments and we show that relapses may be adaptive if they are concomitant with the presence of mosquitoes in the vicinity of the host. We then experimentally test the hypothesis that *Plasmodium* parasites can respond to the presence of vectors. For this purpose, we repeatedly exposed birds infected by the avian malaria parasite *Plasmodium relictum* to the bites of uninfected females of its natural vector, the mosquito *Culex pipiens*, at three different stages of the infection: acute (∼34 days post infection), early chronic (∼122 dpi) and late chronic (∼291 dpi). We show that: (i) mosquito-exposed birds have significantly higher blood parasitaemia than control unexposed birds during the chronic stages of the infection and that (ii) this translates into significantly higher infection prevalence in the mosquito. Our results demonstrate the ability of *Plasmodium relictum* to maximize their transmission by adopting plastic life history strategies in response to the availability of insect vectors.

## Author Summary

Seasonal fluctuations in the environment affect dramatically the abundance of insect species. These fluctuations have important consequences for the transmission of vector-borne diseases. Here we contend that malaria parasites may have evolved plastic transmission strategies as an adaptation to the fluctuations in mosquito densities. First, our theoretical analysis identifies the conditions for the evolution of such plastic transmission strategies. Second, we show that in avian malaria *Plasmodium* parasites have the ability to increase transmission after being bitten by uninfected *Culex* mosquitoes. This demonstrates the ability of *Plasmodium* parasites to adopt plastic transmission strategies and challenges our understanding of malaria epidemiology.

## Introduction

All organisms experience some level of temporal variation in the quality of their environment. In response to these variations, many species have evolved specific strategies that allow them to limit or shut down growth and development until the conditions improve [1]. The best reported examples are dormancy in plants and diapause in insects, but similar strategies have also evolved in microbes. Bacteria can survive adverse conditions (e.g. desiccation, antibiotics) by entering a state of reduced metabolic activity called persistence [2], [3]. Several viruses (e.g. lambdoid phages, herpesviruses) have evolved the ability to integrate their host genome and enter a latent phase during which within-host replication is shut down, the infection is asymptomatic and transmission is very limited [4], [5]. Hence, the evolution of latent life cycle in pathogens may be viewed as an adaptation to temporal variations of the availability of susceptible hosts.

For vector-borne pathogens the abundance of vectors is a key parameter determining the quality of their environment. Vector density may vary in space due to intrinsic heterogeneities of their habitat (e.g. temperature, hygrometry). In malaria, for instance, spatial variation in mosquito abundance has a direct impact on the geographic distribution of prevalence [6]–[8]. Vector abundance may also vary widely through time [9]. Although inter-tropical regions are characterized by a relatively constant density of vectors, regions from higher latitudes experience a broad range of climatic seasonality, and very far from the equator mosquitoes are present for only a fraction of the year [10]–[12]. From the parasite's perspective, such temporal variation in vector density is analogous to the temporal variations in habitat quality experienced by other organisms. How have malaria parasites adapted to these temporal fluctuations in vector density?

Malaria is caused by *Plasmodium* spp., a prevalent vector-borne pathogen which is found infecting many vertebrate hosts, including humans, reptiles and birds. *Plasmodium* infections within the vertebrate host are characterized by drastic temporal changes in blood parasitaemia. After an initial acute phase, generally characterized by a very high number of parasites in the blood, the infection usually reaches a chronic phase where the parasitaemia stabilizes at low levels. During the chronic phase, however, blood parasites may go through short, intense, bouts of asexual replication during which parasitaemia increases temporarily. Little is known about the causes of such *recrudescences* but one potential trigger may be a weakening of the host's immunity [13]. In some, but not all, *Plasmodium* species the infection may entirely disappear from the blood stream, hiding in other host cells in the form of (dormant) exoerythrocytic stages. After a period of latency that can last months or even years, parasites may reappear in the blood stream. These *relapses* are due to the differentiation of dormant parasite stages into new erythrocytic stages. The dormant stages of *Plasmodium* were first described in birds [14], [15] and, later, in humans [16], [17] and reptiles [18], [19]. Relapses and recrudescences have been puzzling researchers ever since the first clinical symptoms were described in *P. vivax*-infected humans in the late 19^th^ century [20], [21]. Why do some malaria species (e.g. *P. falciparum*) completely lack the ability to produce dormant stages in the vertebrate host? What are the ultimate causes of the production of recrudescences and relapses? Is this diversity of life cycles due to the temporal variation in vector density?

The ability to produce recrudescences and relapses may be a genetically fixed parasite strategy that has evolved as a way to match the dynamics of vector populations. Populations exposed to different fluctuations of vector density may thus evolve different strategies. In human malaria, the relapsing periodicity of different lineages of *P. vivax* supports this prediction [12], [22]. Indeed, lineages exhibiting frequent relapses have been sampled in Asia where the vector is present throughout the year. In contrast, long latency has been observed in lineages sampled in temperate zones where the mosquito vector is only present for a few months. In avian malaria, similarly, the differences in the within-host dynamics of *Leucocytozoon spp.* and *Haemoproteus mansoni* may have evolved to match the temporal fluctuations of their respective vector species (simuliid flies and *Culicoides*, respectively) [23].

Another explanation for these patterns may involve adaptive phenotypic plasticity. Phenotypic plasticity is the ability for a single genotype to exhibit different phenotypes in different environments [24], [25]. This contrasts with the above hypothesis (fixed strategy) where different relapsing strategies are associated with different genotypes. The ability to adopt a plastic exploitation strategy requires the ability to detect a change of the environment (i.e. cues) and the acquisition of such a sensing mechanism may be associated with direct fitness costs [24], [25]. In spite of these costs, phenotypic plasticity is often viewed as an adaptation to variable environments [24], [25]. Many pathogens have indeed evolved an unparalleled level of phenotypic plasticity in their life history traits to cope with the temporal variability of their habitat [26]–[28]. In *Plasmodium*, plasticity has been shown to be a response to various stressful conditions such as drug treatment and the presence of competitors [29], [30]. Some experimental evidence suggests that relapses may also be a plastic trait. *P. vivax* relapses are often observed in the spring and summer months irrespective of when the patients got the original infection [31], which suggests that the parasite may react to a change in the physiological state of the host or the environment. Relapses have also been observed in avian malaria, which has triggered several experimental studies to pinpoint the underlying environmental cues [32]. Some authors have proposed that spring relapses may result from increasing photoperiod and/or stress-induced hormonal changes [33]–[36]. Parasites may indirectly benefit from using hormonal and photoperiod cues because they often coincide with (or even anticipate) the appearance of vectors in temperate populations. Such indirect cues are, however, imperfect because vector abundance may be influenced by other, non-seasonal, factors. A more efficient strategy would be to react to direct cues such as mosquito bites which unambiguously indicate the presence of vectors [10], [31], [37]. Although there is some correlational evidence supporting this hypothesis, largely coming from longitudinal cohort studies [10], [37], [38], direct experimental evidence for this hypothesis is scarce and somewhat contradictory. In rodent malaria *P.chabaudi*, mice exposed to probing by *Anopheles stephensi* mosquitoes had higher and earlier parasite growth and gametocytaemia than control unexposed mice [39]. In contrast, however, Shutler *et al.* [40] found no evidence of facultative alteration in the timing or in the level of *P. chabaudi* or *P. vinckei* parasitaemia and gametocytogenesis as a consequence of mosquito probing. Rodent malaria is a laboratory model and, as such, may, not be the best system to test this hypothesis because *An. stephensi* is not the natural vector of rodent malaria [41]. In addition the parasites have been originally sampled from the tropical lowlands of the Congo Basin [42] an area where malaria transmission is high throughout the year [43] and thus the selective pressure for the evolution of plasticity in response to vector availability is expected to be weak. Finally, both rodent malaria experiments [39], [40] were carried out during the initial (acute) phase of the infection, i.e. when parasitaemia is already high (so no need to increase it further) and the infection recent (so the mosquitoes are probably still around). We contend that it is mainly in old (chronic state) infections that the parasite may accrue the greatest benefits from a plastic response to the bites of its vector. Finally, both of these studies used gametocyte density (the blood stages of *Plasmodium* that are transmissible to the vector) as a proxy for transmission but neither followed transmission all the way to the mosquito stage.

**Figure 1:**
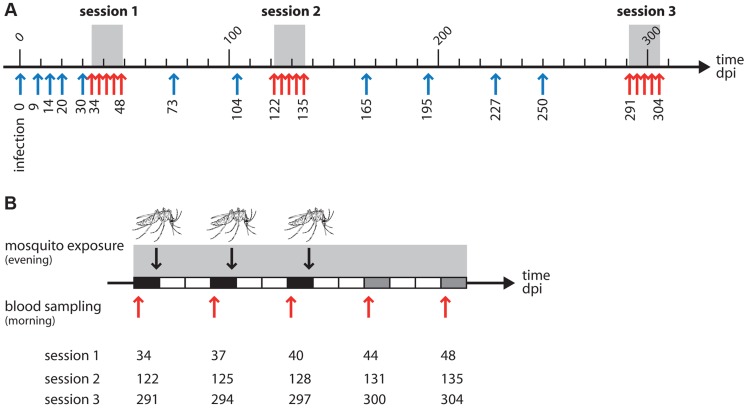
(A) Overview of the experiment with the 3 exposure sessions (grey areas). Arrows indicate the times at which blood samples were taken from the birds: red arrows for the 5 samples taken in and around the time of the mosquito exposure, blue arrows for the regular monitoring of parasitaemia before and between exposures. One unit = 10 days. (B) Zoom on an exposure session: each rectangle represents a day. Black rectangles: blood sample in the morning, mosquito exposure in the evening. Grey rectangles: blood sample in the morning, no mosquito exposure. White rectangles: no mosquito exposure, no blood sampling. Red arrows and figures underneath indicate dates where blood sampling took place in each of the 3 sessions. The mosquito drawing indicates a mosquito exposure.

Here, we first present a theoretical model that studies the evolution of parasite transmission in a variable environment. This model explores the effects of the seasonality of mosquito dynamics on the evolution of virulence and transmission strategies. In particular it clarifies the selective pressures acting on the evolution of temporally variable transmission strategies and identifies the conditions driving the evolution of costly plastic transmission strategies triggered by the exposure to mosquito bites. Then, we carry out an experiment to test the following two hypotheses: (1) *Plasmodium* parasites plastically react to the biting of uninfected vectors by enhancing their within-host replication, and (2) this effect yields higher rates of transmission to the mosquito vector. For this purpose, we studied the interaction between *Plasmodium relictum* (the aetiological agent of the most prevalent form of avian malaria which is commonly found infecting Passeriform birds in Europe) and its natural vector, the mosquito *Culex pipiens*. *P. relictum* is a very convenient malaria parasite to address this issue because it is known to have a long chronic phase marked by sudden events of recrudescences and relapses [44]. Strictly speaking, relapses originate from the division and differentiation of dormant stages (called phanerozoites) that infect the endothelial cells of different organs such as the spleen and liver, while recrudescences originate from an increased replication of the blood stages [44]. In practice, however, it is very difficult to distinguish between recrudescences and true relapses and in the following we will use the term relapse to encompass both cases. We investigate whether bites of uninfected *Cx. pipiens* mosquitoes trigger parasite relapses in the blood of domestic canaries (*Serinus canaria*) chronically infected by *P. relictum* (lineage SGS1), as well as the concomitant effects on transmission in terms of mosquito infectivity (see Box 1 and Fig. 1).

**Box 1. Experimental design**

Twenty birds experimentally inoculated with avian malaria parasite *Plasmodium relictum* were followed for over 300 days post-infection (dpi) to monitor the variation in blood parasitaemia. Birds were either exposed or unexposed (control) to mosquito bites. Exposure to mosquito bites took place in 3 consecutive “exposure sessions” (grey area in figure 1). Each session consisted of 3 “exposure days” separated by 3-day intervals: days 34, 37 and 40 dpi for the first session, days 122, 125 and 128 dpi for the second session, and days 291, 294 and 297 dpi for the third session. In exposure days, each bird in the “exposed” treatment was placed in a cage with a batch of 50 uninfected female mosquitoes for 2 hours; “control” birds were placed in identical conditions but without mosquitoes. Two different response variables were subsequently obtained:

- To estimate changes in blood parasitaemia due to mosquito exposure, blood samples were taken from all (“exposed” and “control”) birds in the morning preceding the exposure as well as 3–4 days and 7–8 days afterwards (days 44 and 48 dpi, days 131 and 135 dpi and days 300 and 304 dpi for exposure sessions one, two and three, respectively). These are indicated by red arrows in Fig. 1. Bird parasitaemia was measured by qPCR on blood samples (see materials and methods for details). Regular monitoring of parasitaemia took place at several other time points before and between each of the exposure sessions (indicated by blue arrows in Fig. 1).
- To estimate the effect of exposure on the prevalence and intensity of mosquito infections, after each exposure blood-fed mosquitoes from all the batches were maintained under standard laboratory conditions for 7 days. Fifteen haphazardly chosen mosquitoes were dissected to check for the presence (prevalence) and number (intensity) of oocysts in the midgut (see materials and methods).

## Results

### Theory: Evolution of plastic transmission strategies

**Figure 2:**
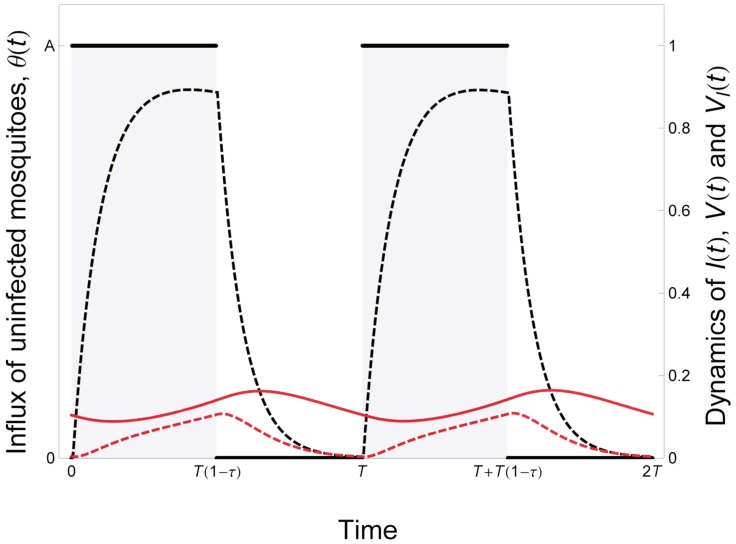
Epidemiological dynamics of a vector-borne disease in a seasonal environment. We consider here that the influx of mosquitoes, *θ*(*t*), is a periodic square wave (see equation (5) in the main text). The parameter *T* measures the duration of the period and the parameter *τ* measures seasonality: the fraction of time where the environment is not suitable for vector reproduction.The epidemiologic dynamics converges to a periodic equilibrium characterised by fluctuations of the uninfected and infected vector the densities: *V*(*t*) and *V_I_*(*t*) (black and red dashed lines, respectively). We also plot the dynamics of the density of infected hosts: *I*(*t*)/10 (red line). Parameter values: 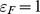, 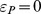, 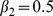, 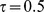. See default values in the Text S1 for other parameters.

To model the evolution of plastic transmission strategies we first need to model the epidemiological dynamics of malaria. For the sake of simplicity the vertebrate host population is assumed to be constant and equal to *N* = *S*(*t*)+*I*(*t*), where *S*(*t*) and *I*(*t*) are the densities of uninfected and infected hosts, respectively. In contrast, the density of the vector population may change through time. This may be particularly relevant in temperate environments where the mosquitoes do not reproduce in winter. In other words, the influx *θ*(*t*) of uninfected mosquitoes is assumed to change throughout the year (i.e. *θ*(*t*) fluctuates with a period *T*). As a consequence, the densities *V*(*t*) and *V_I_*(*t*) of uninfected and infected vectors also fluctuate through time. The following set of differential equations describes the temporal dynamics of the different types of hosts (the dot notation indicates differential over time):
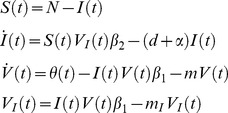
(1)Where *d* is the natural mortality rate of the vertebrate host and *α* is the virulence of malaria (the extra mortality induced by the infection); *m* and *m_I_* are the mortality rates of uninfected and infected vectors, respectively; *β_1_* is the transmission rate from the vertebrate host to the vector; *β*_2_ is the transmission rate from the vector to the vertebrate host. Figure 2 presents a typical epidemiological dynamics in a seasonal environment.

What are the consequences of the temporal variation in the availability of vectors on the evolution of malaria? More specifically, what is the effect of the shape of the function *θ*(*t*) on the evolution of the parasite? To study this question one can consider the fate of a mutant malaria strategy *M* that would alter its life history strategy in the vertebrate host. The replication in the vertebrate host is assumed to be governed by two traits of the parasite. The first trait, *ε_F_*, governs the allocation to a *fixed* exploitation strategy that yields a within-host growth rate that does not vary with time. In contrast, the second trait, *ε_P_*, governs allocation to a *plastic* exploitation strategy that may vary with time. More specifically we consider that when the parasite adopts this plastic trait within-host growth rate depends on the density of vectors in the population. In other words this plastic trait allows within-host growth rate to be conditional on the presence of vectors. Because within-host growth rate is assumed to affect virulence in the vertebrate host this yields:
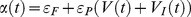
(2)


As in classical models of virulence evolution [45], [46] more replication is costly because it may induce the death of the vertebrate host but it allows the parasite to transmit more efficiently. The parameters *a* and *b* govern the specific shape of the virulence-transmission trade-off (see equation (3) below). In addition we assume that the adoption of a plastic exploitation strategy requires the ability to acquire information regarding the availability of the vectors. The parameter *c*, therefore measures the fitness cost associated with a higher investment in the mechanisms allowing such plasticity. Only the transmission rate, *β*_1_, from the vertebrate host to the vector is assumed to be affected by the parasite exploitation strategy (i.e. β_2_ is assumed to be constant) which yields:
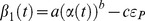
(3)Note that in this model virulence and transmission vary in time only if the parasite allocates some resources in the development of a plastic trait (i.e. *ε_P_*>0).

Integrating the change in frequency of the mutant parasite genotype *M* over one period of the fluctuation allows deriving a condition for the invasion of the mutant (see Text S1):
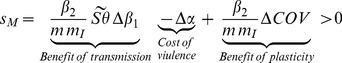
(4)where 
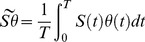
, 
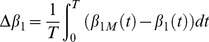
, 
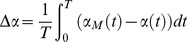
 and 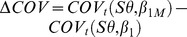.

The first term in the above equation for *s_M_* is the classical benefit associated with higher investment in transmission. If the mutant invests more than the resident in transmission (i.e. Δ*β*_1_>0) the fitness increase depends on 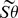, which measures the availability of both uninfected hosts and vectors over the period of the fluctuation of the environment. The second term in *s_M_* is the classical cost of virulence if the mutant exploits the host more aggressively than the resident (i.e. Δ*α*>0). The final term in *s_M_* measures the potential benefit associated with plastic transmission strategies. This term depends on the covariance between the availability of uninfected vectors, the availability of uninfected vertebrate hosts and the investment in transmission from vertebrate to mosquito hosts. The mutant may gain a fitness advantage if its conditional transmission rate can better track the fluctuations of the density of uninfected hosts. In other words this final term indicates that in a fluctuating environment it is adaptive to invest on transmission whenever uninfected hosts and mosquitoes reach high densities simultaneously. We can use this analysis to look at different evolutionary scenarios.

#### Without plasticity

**Figure 3:**
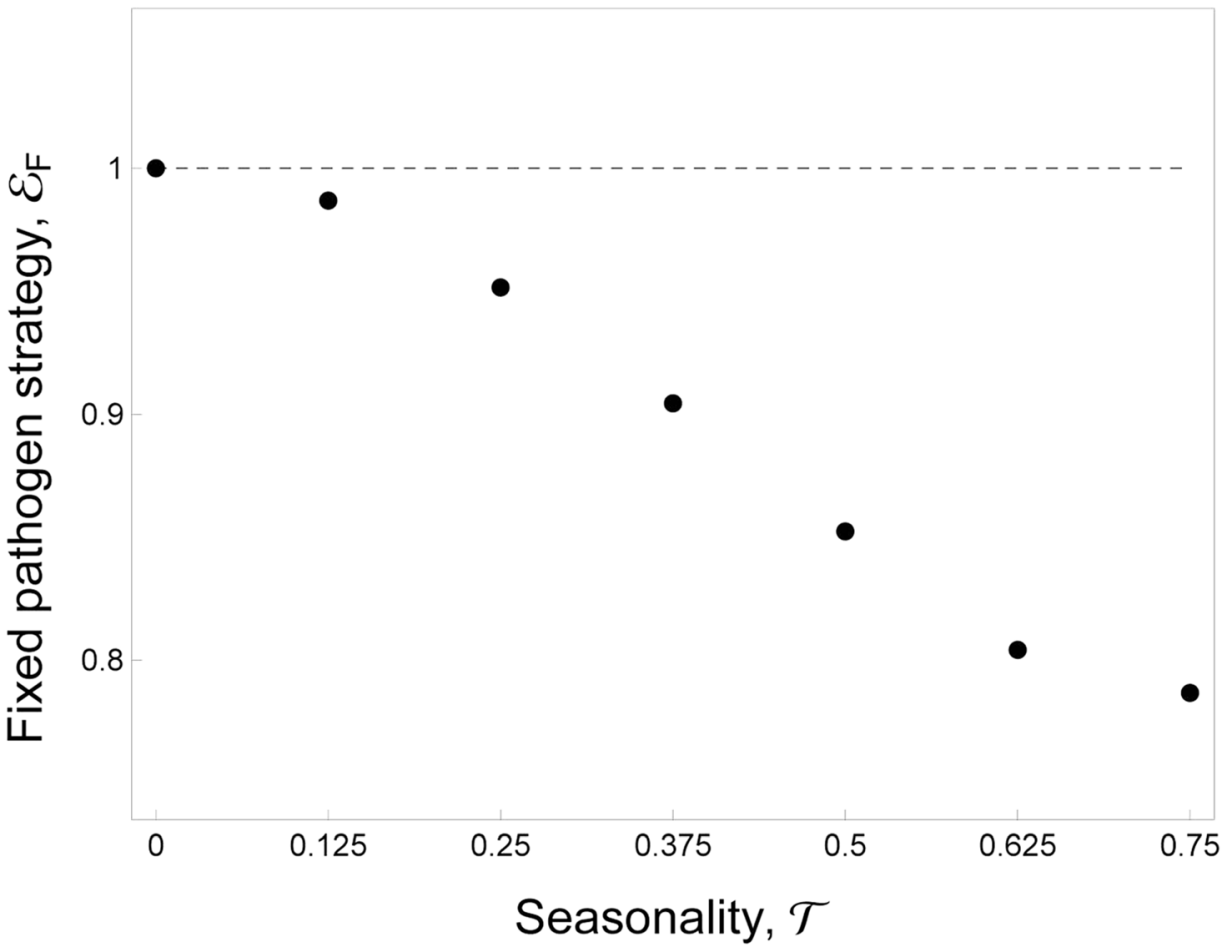
Evolution of allocation to fixed pathogen strategy, *ε_F_*, as a function of seasonality, *τ*. A higher investment in *ε_F_* indicates that the pathogen invests more in transmission (and virulence). Parameter values: *ε_P_* = 0. See default values in the Text S1 for the other parameters.

For the sake of simplicity let us focus first on the influence of seasonality on the evolution of the fixed exploitation strategy in the absence of plasticity. In this case *ε_P_* = 0 and neither virulence nor transmission vary in time. Consequently the final term in *s_M_* drops and evolution is driven by the balance between the benefit of transmission and the cost of virulence. In our model the cost of virulence is not affected by the epidemiological dynamics (infected hosts die because of the infection irrespective of the presence of the vectors). In contrast, the benefit of transmission is weighted by the quantity 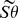 which measures the overall opportunity of contacts between uninfected hosts and vectors. Fluctuations in *θ* over time may not necessarily affect the quantity 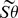 and in these situations seasonality has no impact on virulence evolution (Text S1). Yet, contrasting situations with or without wintering season dramatically affects the average influx of vectors and consequently the quantity 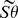. To study the effect of seasonality we model the fluctuations of the influx of uninfected mosquitoes as a periodic square wave (Fig. 2):
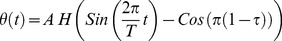
(5)Where *A* is the influx of uninfected mosquitoes when the environment is favorable for mosquito reproduction, *T* is the period of the fluctuations, *τ* is the fraction of time unsuitable for mosquito reproduction and *H*(*x*) is the discontinuous unit step function taking the value 0 (when *x*<0) or 1 (when *x*>0). In Figure 3 we show that when seasonality increases the quantity 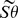 drops, the benefit of transmission is reduced and the parasite evolves toward lower virulence and lower transmission rates. In other words our model predicts that, away from the tropics, malaria population will experience more seasonal environments and the investment in the fixed level of host exploitation drops to avoid the cost of virulence when no vectors are around. This should yield lower levels of transmission and virulence in higher latitudes.

#### With plasticity

**Figure 4:**
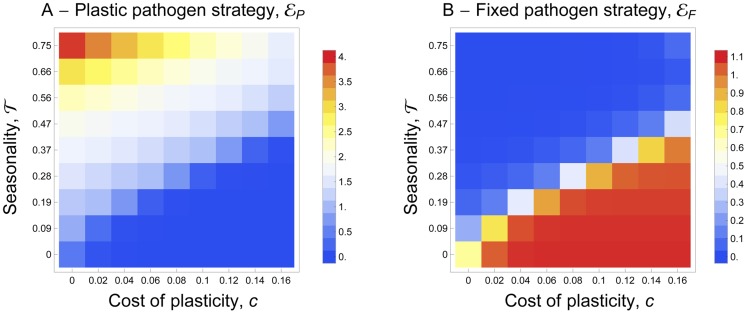
Joint evolution of (A) the plastic pathogen strategy, *ε_P_* and of (B) the fixed pathogen strategy, *ε_F_* for different values of seasonality, *τ*, and for different costs of plasticity, *c*. The color shading indicates the value of the pathogen strategies and the warmer color indicates higher values. A higher investment in *ε_P_* indicates that the pathogen invests more into the mechanisms that allow it to react to the presence of mosquitoes. A higher investment in *ε_F_* indicates that the pathogen invests more into transmission (and virulence). For both strategies the lower value (blue) is 0. The maximal value (red) of *ε_P_* is 4 and the maximal value (red) of *ε_F_* is 1.1. See default values in the Text S1 for the other parameters.

Our model can also be used to understand the conditions leading to the evolution of plastic transmission strategies. In this case we allow both fixed and plastic exploitation strategies (i.e. *ε_F_* and *ε_P_*) to evolve freely. Figure 4 shows that when the vectors are present throughout the year the pathogen does not invest in a costly plastic strategy. In this case, the parasite population evolves toward the fixed level of host exploitation studied in the previous scenario and resulting from the balance between the benefit and the cost of parasite virulence. When the environment becomes more seasonal (i.e. *τ*>0) the allocation to a fixed exploitation strategy drops and the pathogen invests in the costly plastic strategy (Fig. 4). The plastic strategy allows allocating resources to host exploitation only when vectors are around. Hence this relatively simple model confirms that a costly plastic transmission strategy that depends on the availability of vectors in the environment can outcompete a constant exploitation strategy. Note that for intermediate levels of seasonality the evolutionarily stable exploitation strategy is a mixture between a constant and a plastic strategy.

### Experiment with avian malaria

The experimental design is presented in Box 1. In brief, we followed 20 experimentally infected birds over 300 days post infection and monitored within-host parasitaemia and transmission to vectors. Birds were assigned to two treatments: “exposed” or “control” (unexposed) to uninfected mosquito bites during 3 sessions (starting 34, 122 and 291 days post infection, see Fig. 1A). During each session the exposed birds were bitten by a batch of 50 female mosquitoes every 3 days (see Fig. 1B).

#### Parasitaemia

**Figure 5:**
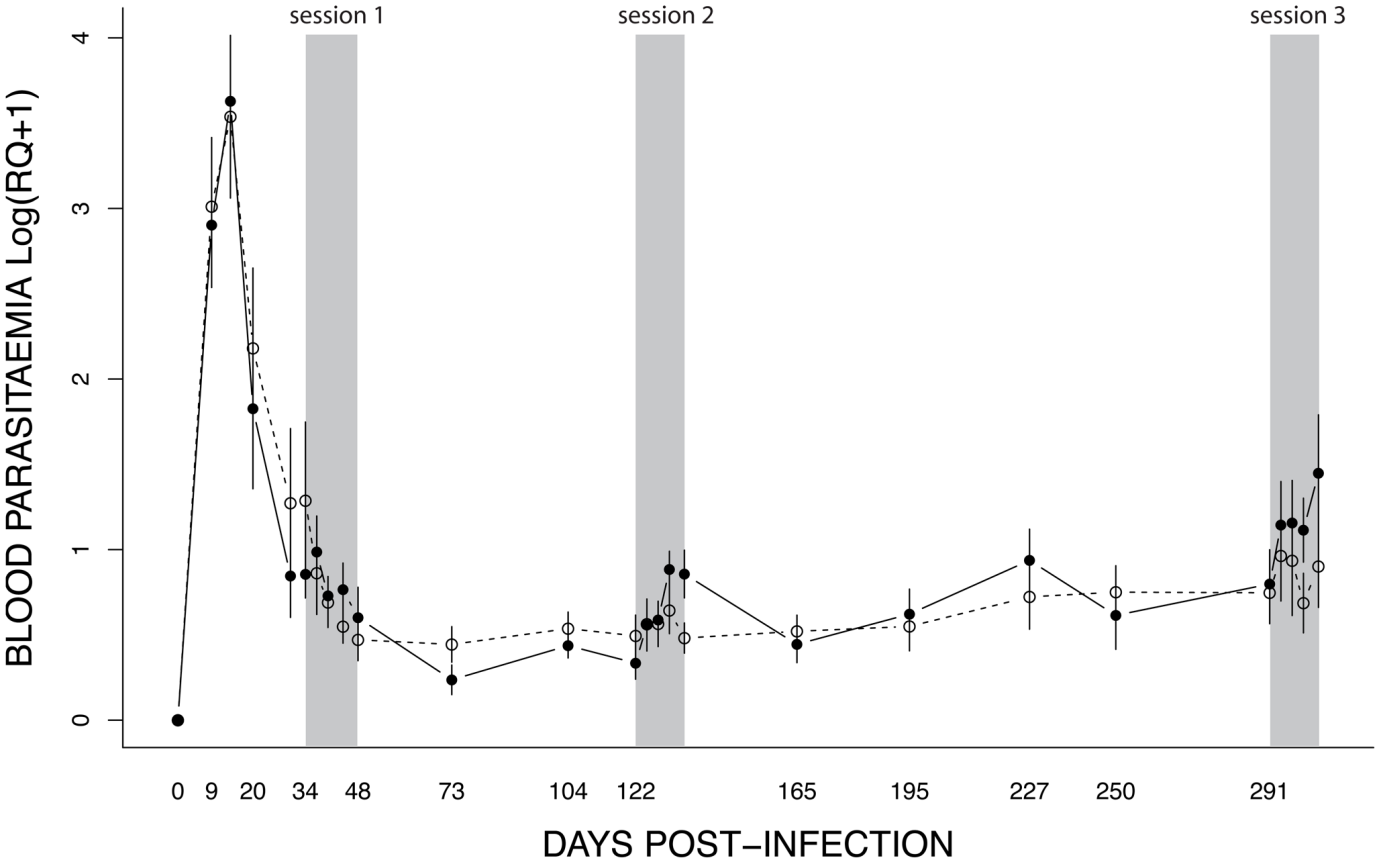
Dynamics of blood parasitaemia (Log(RQ+1), mean ± s.e.) of *Plasmodium relictum* (lineage SGS1) in birds that were either unexposed (open circles, dashed line) or exposed to mosquito bites (filled circles, solid line). Mosquito exposure (refer to Materials & Methods for details) took place in three consecutive sessions (grey areas): 34–48 dpi, 122–135 dpi and 291–304 dpi.

The parasitaemia initially followed a bell-shape function typical of acute *Plasmodium* infections: peaking at day 14 pi and decreasing thereafter (Fig. 5). The infection subsequently entered a long-lasting chronic state, which was characterized by a low (but detectable) blood parasitaemia over several months (Fig. 5).

**Figure 6:**
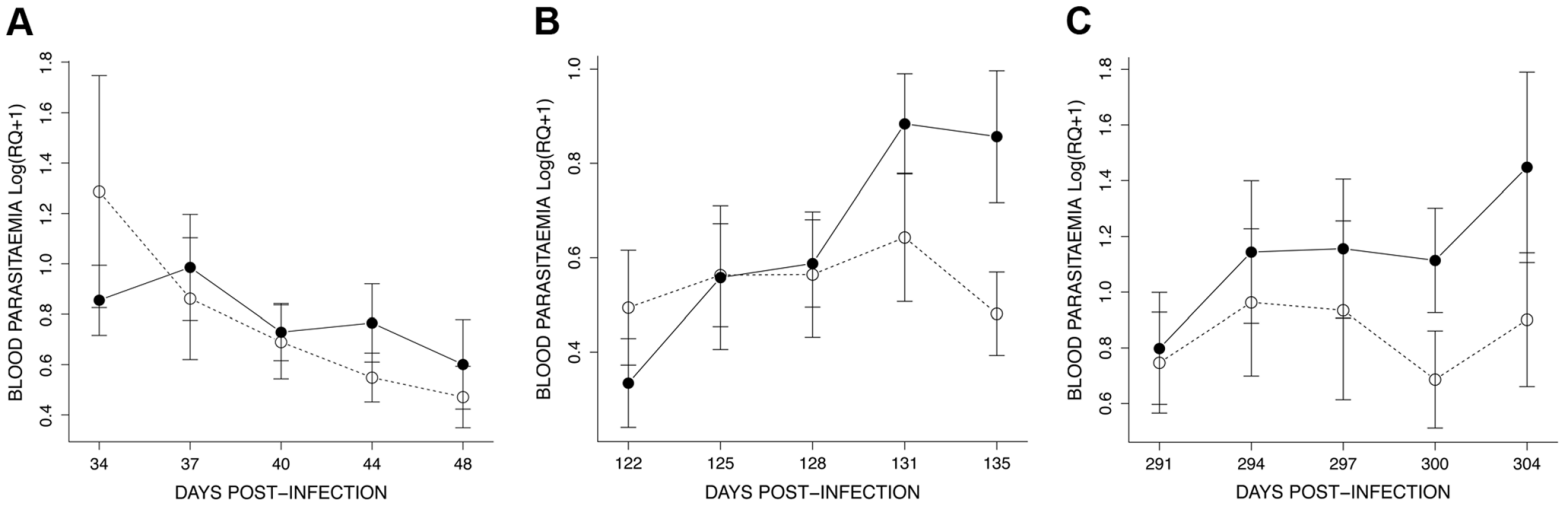
Details of the dynamics of blood parasitaemia (Log(RQ+1), mean ± s.e.) for the 3 exposure sessions (see Fig. 4): (A) session 1 (34–48 dpi), (B) session 2 (122–135 dpi) and (C) session 3 (291–304 dpi). Unexposed (open circles, dashed line) or exposed to mosquito bites (filled circles, solid line).

The effect of mosquito exposure on blood parasitaemia was analysed separately for each of the 3 exposure sessions. In the first (34–48 dpi) session, parasitaemia was still decreasing after the initial (acute) phase (*time* effect: χ^2^_1_ = 11.14, *P* = 0.0008) but this decrease was independent of whether the birds had been exposed to mosquitoes or not (*exposure* effect: χ^2^_1_ = 0.007, *P* = 0.9345) (Fig. 6A). In the second (122–135 dpi) session, however, bird parasitaemia differed between the exposed and the unexposed (control) birds. Whereas in the control birds the total number of parasites remained roughly constant with time, parasitaemia in the exposed group increased significantly with time (exposure*time: χ^2^_1_ = 9.18, *P* = 0.0024, Fig. 6B). In the third (291–304 dpi) session, parasitaemia showed a similar trend towards a higher parasitaemia in exposed mosquitoes (Fig. 6C), but this trend was not statistically significant (*exposure*: χ^2^_1_ = 0.40, *P* = 0.5270; *time*: χ^2^_1_ = 18.87, *P* = 0.0003). At this time point, however, several birds had died, which reduced the statistical replication and limited the statistical power of the test (*n* = 6 birds alive on day 291, *n* = 3 and *n* = 4 on day 304 for exposed and control birds, respectively) (see Table S1 for group sample sizes).

It is worth noting that the effect of exposure to mosquito bites seems to be short-lived. Indeed, one month after the second exposure session (165 dpi) we did not detect any difference in parasitaemia between the exposed and the control birds (χ^2^_1_ = 0.03, *P* = 0.5932, see Fig. 5).

#### Mosquito infection

**Figure 7:**
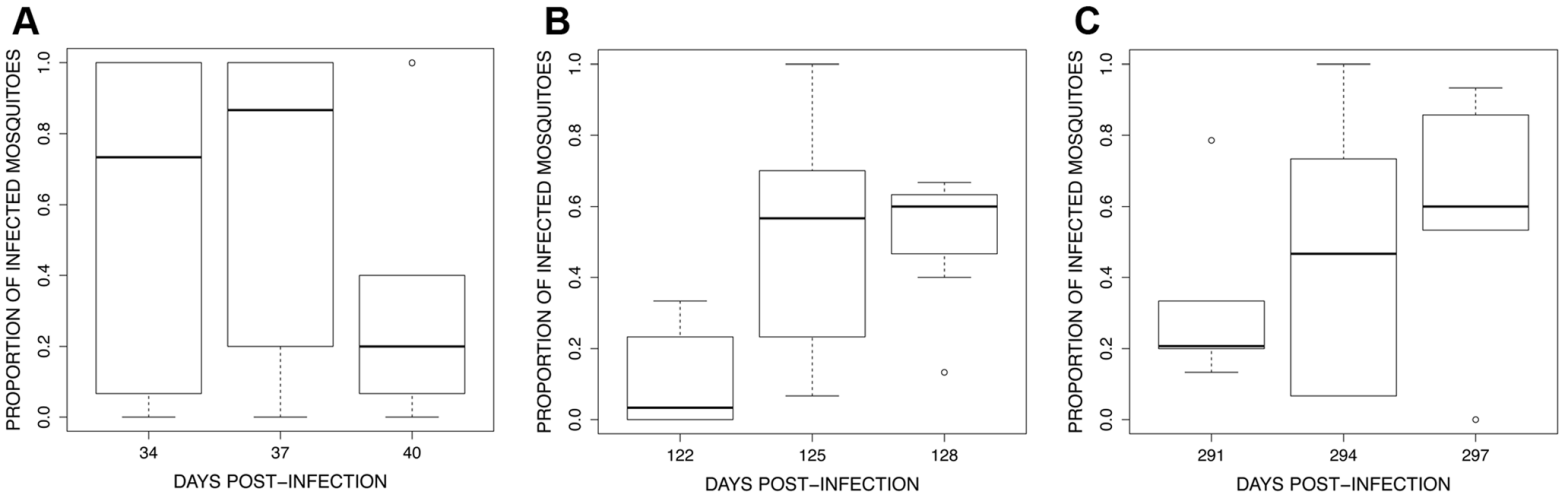
Boxplot of the proportion of infected mosquitoes among 15 haphazardly chosen blood fed individuals on each bird (harbouring at least 1 oocyst in the midgut) for the 3 exposure sessions (see Fig. 4): (A) session 1 (34–40 dpi), (B) session 2 (122–128 dpi) and (C) session 3 (291–297 dpi). The figure shows the median proportion of infected mosquitoes (horizontal black bars). The white boxes below and above the median indicate the first and third quartiles respectively. Dashed lines delimit 1.5 times the inter-quartile range on both side of the box, above which individual counts are considered outliers and marked as dots.

In the first exposure session, infection prevalence (proportion of mosquitoes containing at least 1 oocyst) was extremely high among the first batch of mosquitoes (Table S2) but decreased in subsequent batches (contrast 34+37*vs.* 40 dpi, χ^2^_1_ = 51.71, *P*<0.0001; Fig. 7A). This effect is linked to the decrease in overall parasitaemia (χ^2^_1_ = 48.45, *P*<0.0001) and is likely due to the fact that the first session occurred at the end of the acute phase and before the start of the chronic phase (see Fig. 5). In contrast, in both the second and third exposure sessions infection prevalence increased significantly in successive mosquito batches (second exposure session: contrast 122 *vs.* 125+128 dpi, χ^2^_1_ = 66.34, *P*<0.0001; third exposure session: χ^2^_2_ = 25.99, *P*<0.0001, here all time points differed from each other, Fig. 7B and B).

**Figure 8:**
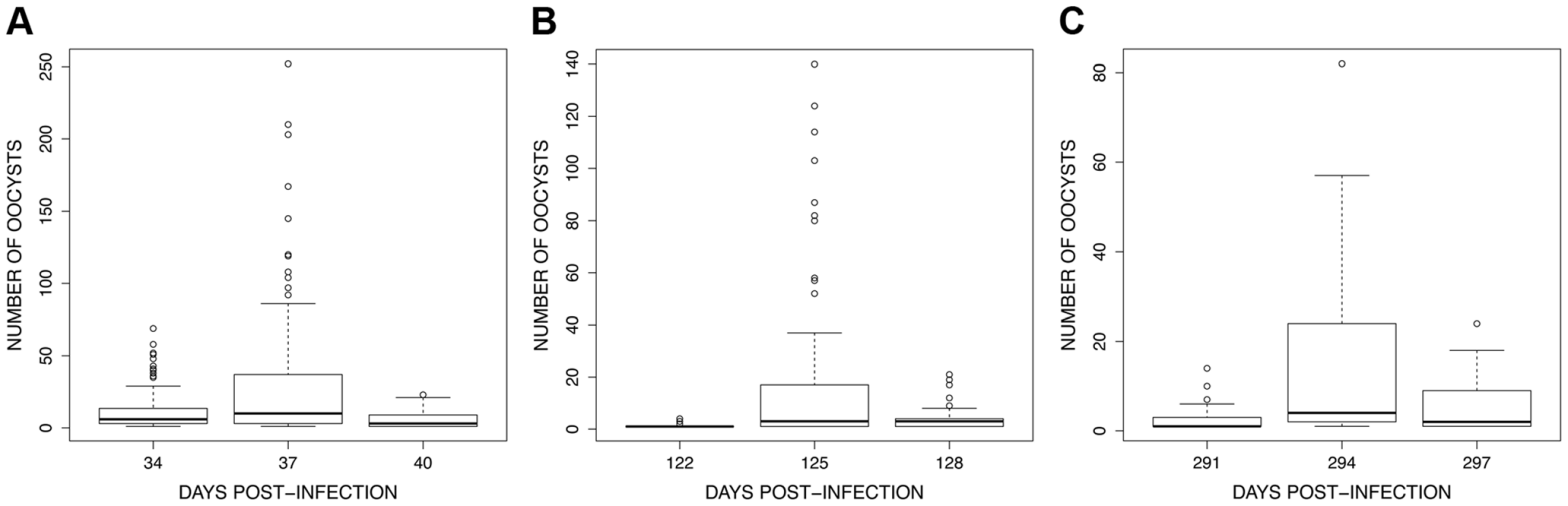
Boxplot of the number of oocysts per midgut among 15 haphazardly chosen blood fed individuals on each bird (only includes mosquitoes harbouring ≥1 oocysts) for the 3 exposure sessions (see Fig. 4): (A) session 1 (34–40 dpi), (B) session 2 (122–128 dpi) and (C) session 3 (291–297 dpi).

The analysis of oocyst burden only included mosquitoes having one or more oocysts in the midgut. Oocyst burden showed a consistent pattern across the three exposure sessions: the number of oocysts increased significantly between the first and second mosquito batches but decreased thereafter (*batch time* effect: χ^2^_2_ = 1147, *P*<0.0001, χ^2^_2_ = 546.17, *P*<0.0001 and χ^2^_2_ = 389.84, *P*<0.0001 for the first, second and third exposure sessions respectively; all contrast analyses were significant; Fig. 8).

To verify that the control birds were still infective to mosquitoes, they were exposed to mosquitoes on day 307 pi. Only three birds survived to this point and all three were infective to mosquitoes (Table S2).The infection rate of mosquitoes biting the control birds was compared to the infection rate of the last batch of mosquitoes biting the exposed birds (297 dpi). As expected from the above results, the infection rate of mosquitoes biting a bird for the first time was significantly lower than the infection rate of mosquitoes biting a bird which has been recently bitten by two batches of mosquitoes in previous days (infection rate of mosquitoes biting the control birds: 0.36±0.12, exposed birds 0.58±0.10; χ^2^_1_ = 5.76, *P* = 0.0164).

## Discussion

Plasticity has evolved as an adaptation to the variability of the environment in many organisms [25], [47], including pathogens [27], [28], [48], [49]. Here we contend that the evolution of fixed or plastic dormancy strategies in *Plasmodium* may be an adaptation to the seasonal fluctuations of vector densities. We explore this hypothesis with a theoretical model and test experimentally some of our predictions in avian malaria.

### Theory

**Figure 9:**
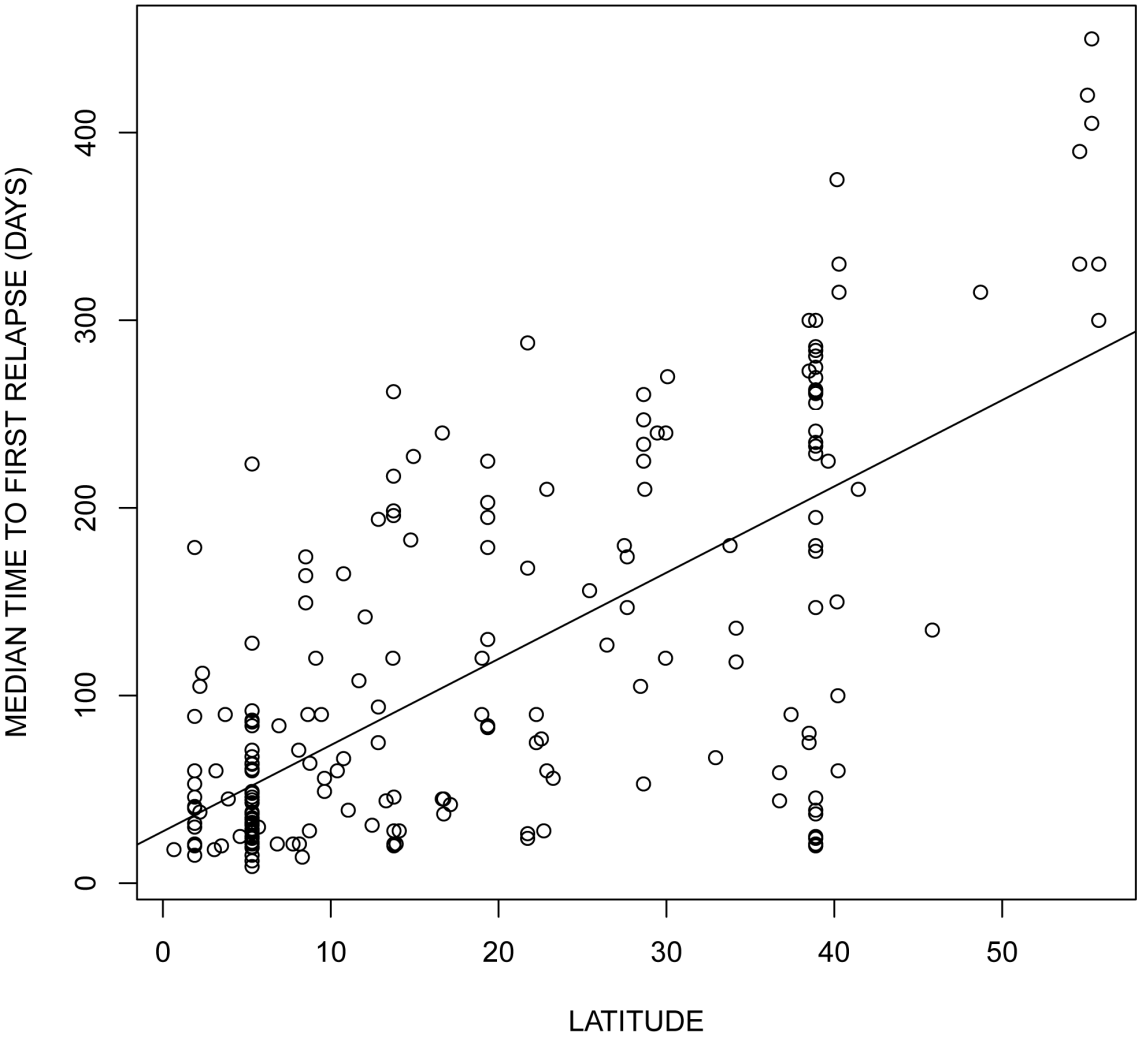
Effect of latitude on the relapsing rate of *Plasmodium vivax*. The data was obtained from the supplementary Table S1 of Battle *et al.* [22]. Each dot represents a parasite strain originating from different locations. The latitude of origin has a significant effect on the observed time to first relapse (R^2^ = 0.4966, *F*_1,232_ = 228.9, *P*<0.0001).

How do malaria parasites adapt to the density fluctuations of their insect vectors? To answer this question we started by studying the evolution of transmission strategies using a classical epidemiological model for a vector-borne pathogen. This theoretical approach helps clarify the multiple effects of temporal fluctuations of vector populations. We first considered the evolution of a fixed allocation to virulence and transmission. Our analysis shows that the effect of the temporal variation is driven by its effect on the average density of susceptible hosts and vectors over one period of the fluctuation. In particular we show that in more seasonal environments (e.g. higher latitudes), where the vectors can pullulate only for a few months, lower levels of virulence and transmission should be selected. This is because, in our model, seasonality reduces the average number of vectors. In the absence of the vector, investing in transmission becomes maladaptive because within-host reproduction is associated with higher virulence and host death. This result is very similar to the effect of periodic host absence on the evolution of phytopathogens when there is a trade-off between pathogen transmission and pathogen survival [50]. In addition, our predictions agree with recent models studying the effect of seasonality on virulence evolution [51], in that if the fluctuations of vector density do not affect the mean density of susceptible vectors over time, we expect no evolutionary consequences. Interestingly, our prediction on the effect of seasonality (Fig. 3) is consistent with the geographical distribution of relapsing strategies in *P. vivax* [22]. *P. vivax* genotypes sampled near the equator (where seasonality is minimal) invest in higher transmission strategies (higher rates of relapse) than *P. vivax* genotypes sampled in higher latitudes. In other words, in *P. vivax* malaria latitude is a very good predictor of the rate of relapses (Fig. 9).

In a second step of the analysis we allowed plastic transmission strategies to evolve. In particular, we assumed that the malaria pathogens have the ability to sense the density of vectors through exposure to mosquito bites. We derived the condition promoting the evolution of such plastic behaviours when investment in this strategy is associated to a direct fitness cost on transmission. Koelle *et al.* [52] derived a similar result in a model of pathogen adaptation to seasonal fluctuations but without highlighting the force driving adaptive plasticity. Kumo and Sasaki [53] showed that the sensitivity to seasonality in a directly transmitted pathogen is driven by the correlation between the seasonal variation in transmission rate and the density of susceptible hosts. In our model the sensitivity to seasonality is governed by the fluctuation of mosquito density and plasticity. Similarly we show that what selects for plasticity is the covariance between transmission and the availability of hosts (both the vertebrate hosts and the vectors). In other words, plasticity evolves when mosquito bites provide accurate information on the availability of susceptible hosts. Cohen [54] obtained very similar results on the evolution of conditional dormancy strategies in randomly varying environments. The evolution of conditional dormancy depends on the correlation between the cue and the quality of the environment for individuals leaving the dormant state [54] (see also [55], [56]). In our model the correlation between the cue (mosquito bites) and the abundance of susceptible hosts depends on seasonality: as expected, plasticity evolves more readily when mosquitoes are only around for a short period of time.

### Experiment

Have malaria parasites evolved the ability to respond plastically to mosquito bites on its vertebrate host? Previous work on acute rodent malaria infections has produced somewhat contrasting results [39], [40]. These earlier studies had in common that (i) they used an unnatural mosquito-*Plasmodium* combination, (ii) they were carried out using parasites collected in a high-transmission tropical environment several decades ago and (iii) were carried out when the infection is already at its highest level within the vertebrate host. Here we use a natural mosquito-*Plasmodium* combination to test the effect of mosquito bites on parasite transmission during the chronic phase of the infection. We used a *P. relictum* lineage (SGS1) which had been sampled from wild house sparrows in 2009 in a high latitude habitat (Dijon, France) where the environment is characterized by marked seasonal patterns, including variations in mosquito prevalence [57]. In addition, rather than inferring parasite transmissibility solely from the host's parasitaemia, we also quantified the number of parasites that made it all the way to the gut (oocyst) stages of the biting mosquitoes. Our experiment confirmed our two main predictions. First, *P. relictum* SGS1 reacts to mosquito bites by increasing its overall parasitaemia in the blood. As expected, this effect was not present during the acute infection (first exposure session) because transmission is always high at this stage, but became apparent during the chronic stage of the infection (second and third exposure sessions). Second, this increased parasitaemia resulted in higher probability of infection to mosquitoes and thus in higher transmission rates. The results were consistent at the chronic stage of the infection (exposure sessions 2 and 3): there was a significant increase in mosquito infection rate after exposure to mosquito bites.

Blood stage malaria infections comprise both asexual (replicating) and sexual (transmissible) stages. However, the molecular tools used to quantify overall parasitaemia in this study did not allow us to distinguish between these two parasite life stages. In other malaria systems the conversion rate between the asexual and the sexual (gametocyte) stages, and the resulting sex ratio of the gametocytes may be highly plastic [30], [58], so that overall parasitaemia may not necessarily be a good predictor of gametocyte density and/or transmission. Although nothing is yet known about the conversion rates or sex allocation strategies in *P. relictum*, in our experiment the increase in parasitaemia was accompanied by a significant increase in the number of infected mosquitoes, suggesting a concomitant increase in gametocyte density in birds exposed to mosquito bites. However, to directly test this hypothesis, we compared the gametocytaemia of exposed and unexposed birds by counting the visible gametocytes in the thick blood smears taken after the exposure (see Text S2). Contrary to expectations, however, we found no clear and consistent evidence that mosquito bites result in higher gametocytaemia. One potential explanation of this lack of consistency is an error in our estimate of gametocytaemia. The application of molecular techniques for the quantification of gametocytes has indeed called into question the use of microscopic methods to quantify *Plasmodium* gametocytes [59]. In particular, these studies have shown that submicroscopic gametocyte densities are common [60], [61] and can readily infect mosquitoes [62]. Unfortunately these molecular tools are currently only available for *P. falciparum and P. chabaudi*, and no equivalent tools exist to estimate gametocytaemia in *P. relictum*.

A potential caveat of these results is that all the mosquitoes used in the same exposure session emerged roughly at the same date. As a consequence, females from the second and third mosquito batches were 3 and 6 days older (respectively) than mosquitoes used in the first batch. To control for a potential confounding effect of female age on transmission we therefore carried out another experiment using females of identical age (7 days old) at each exposure session. This experiment was carried out using laboratory (SLAB strain) mosquitoes and although the oocyst infection intensities were overall lower, we obtained qualitatively similar effects as in the main experiment (see Text S3). In addition, earlier studies have found either that age has little effect on mosquito vector competence [63] or that older mosquitoes have a lower prevalence and intensity of infection than their younger counterparts [64]. Hence, the increase in oocyst prevalence and intensity observed in consecutive exposure sessions in our main experiment cannot be explained by differences in the age of the mosquitoes used.

### Mechanisms

The proximal mechanism governing this form of plasticity remains to be investigated. How do parasites in the blood or in tissues perceive mosquito bites? A plethora of substances and molecules present in the salivary fluid are injected when mosquitoes probe and feed [65]. The primary role of these molecules is to combat host homeostasis and to regulate inflammation at the biting site to facilitate blood uptake. Vector salivary lysates have been shown to stimulate within-host growth of *Leishmania* parasites [66] and may also trigger plastic life-history strategies in *Plasmodium*. In addition, host anaemia, erythropoeisis, and asexual density have all been shown to be associated with the onset of gametocytogenesis in rodent malaria [67]–[70]. Shutler *et al.* [40] suggested that blood feeding mosquitoes may cause host anaemia thereby triggering gametocytogenesis in *P. chabaudi*. Our data, however, do not support this hypothesis, because birds exposed to mosquitoes had similar or even higher haematocrit than control birds (see Text S3). In addition, previous findings obtained using *P. gallinaceum* have shown that in this avian malaria parasite, parasitaemia and gametocytaemia are not affected by host anaemia [71]. The study of the mechanisms governing plastic transmission strategies in avian malaria is hampered by the lack of available molecular tools to quantify and sex gametocytes (e.g. [72] for rodent malaria). Mechanistic studies can reveal fascinating pathogen strategies. For instance, a recent study on the Cauliflower mosaic virus (CaMV) has shown that when aphids feed on the infected plants the virus reacts instantly (and reversibly) to maximize its transmission. For this purpose it modifies the distribution of a specialised set of proteins which are essential for virus transmission [73]. In the absence of the vector, these proteins, which are toxic for the plant, are neatly packed away inside specialised structures called “transmission bodies”. This study not only represents an excellent example of the ability of some vector-borne pathogens to adopt plastic transmission strategies but it also demonstrates the sophisticated molecular and cellular mechanisms that may be involved.

### Perspectives

#### Theory

The theoretical framework we developed could be used to consider other forms of fluctuations of the environment. For instance, at a short time scale, most malaria parasites show a periodicity in replication burst and allocation to parasite transmissible stages. The synchronicity between this periodicity and the fluctuation of vector density is very important for transmission success [74] and Hawking postulated that the periodicity of malaria may have evolved as a way to maximize the availability of mature gametocytes when mosquitoes feed [75]. Although this hypothesis remains controversial [76], [77] our approach could help identify the conditions that may promote the evolution of cell cycle coordination in malaria as a response to daily fluctuations of vector availability.

Our theoretical framework could be readily extended to consider the evolution of other cues triggering higher transmission rates. In the field, co-infections of different *Plasmodium* strains or species are the norm rather than the exception [78], [79]. The relapsing strategy of malaria may depend on the presence of other competing pathogens within the host [80]. It would be particularly interesting to investigate the effect of coinfections on the activation of relapses [81], [82].

#### Experimental

Our model predicts that seasonality could have a huge impact on the evolution of pathogens (see Fig. 4). Figure 9 shows data in *P. vivax* supporting our prediction on the evolution of fixed pathogen strategies. Testing our predictions regarding the effect of seasonality on the evolution of plasticity may, however, be more challenging. The well recorded worldwide distribution of avian malaria (MalAvi Database, [83]), however, provides an unparalleled opportunity to test this hypothesis. We predict that tropical lineages should exhibit lower potential for plasticity and lower response to external stimuli (such as mosquito bites) than temperate lineages.

It is important to investigate further the role of the quantity and the quality of the mosquitoes on the reactivation of dormant parasites. Quantity-wise, the threshold number of mosquitoes required for the reactivation to take place should be established. Billingsley *et al.* [39] reported an effect of the amount of mosquitoes biting on gametocytaemia of rodent malaria. Quality-wise, it would be interesting to study if plasticity is specific to particular vector species. Indeed, we may expect *Plasmodium* to respond only to the bites of mosquitoes species that serve as competent vectors of the disease [66]. Alternatively, a general response to the bites of non-vector species may be indicative of an evolutionary constraint (the inability of the parasite to distinguish between vector and non-vector signals), or of a temporal correlation in the abundance of vector and non-vector species in a given area.

### Conclusions

We identified the conditions that promote the evolution of plastic transmission strategies in a fluctuating environment. In line with our theoretical predictions, we show that *P. relictum* has the ability to boost its own transmission during the chronic phase of the vertebrate infection after being exposed to mosquito bites. Whether this ability extends to other *Plasmodium* species and in particular to human malaria remains to be investigated. In *P. vivax* the data presented in Figure 9 indicates a strong effect of latitude (i.e. seasonality) on relapses and transmission. The role of plastic transmission strategies on this pattern is unclear but it deserves further investigation. This may help define better control strategies, with more specific recommendations on both spatial and temporal implementations of targeted interventions against malaria hotspots [84]. The study of plastic transmission strategies may also be relevant to many other pathogens that are known to alternate between acute and dormant phases such as varicella zoster virus [85] Herpes Simplex virus [86], *Mycobacterium tuberculosis* [87] and HIV [88]. Such dormant parasites pose considerable therapeutic challenges and much would be gained from understanding the cues underlying the switch between dormant and acute stages in these pathogens [89]–[91]. In conclusion, a better understanding of the ecological determinants as well as the evolutionary forces governing parasite relapses is not only of academic interest: it is also urgently needed to improve the efficacy of public health strategies.

## Materials and Methods

### Ethics statement

Animal experiments were carried out in strict accordance with the National Charter on the Ethics of Animal Experimentation of the French Government, and all efforts were made to minimise suffering. Experiments were approved by the Ethical Committee for Animal Experimentation established by the authors' institution (CNRS) under the auspices of the French Ministry of Education and Research (permit number CEEA- LR-1051).

### Malaria parasites and mosquitoes

*Plasmodium relictum* (lineage SGS1) is a generalist parasite and the most prevalent form of avian malaria in Europe, infecting over 30 birds species in the order Passeriformes [44], [83]. Our strain was originally isolated from wild house sparrows caught in the region of Dijon (France) and maintained in the laboratory via passages to naïve canaries either by intraperitoneal injection or through the bite of infected *Culex pipiens* mosquitoes.

Experiments were conducted with wild *Cx. pipiens pipiens* mosquitoes. *Cx. pipiens* is the natural vector of *P. relictum* in the wild [44], [92], [93]. Thousands of L3 and L4 larvae were collected from a single sewage treatment lagoon in the village of Triadou (20 km north Montpellier, France) using a hand net and reared till adulthood under standard laboratory conditions [94]. We used females 7, 10 and 13 days after emergence that had had no prior access to blood, had been maintained on glucose solution (10%) since their emergence, and had been starved (but provided with water) for 6 h before the experiment.

### Experimental design

Experiments were carried out using (1-year old) domestic canaries (*Serinus canaria*). Prior to the experiments, a small amount (*ca.* 15–25 µL) of blood was collected from the brachial vein of each of the birds and used for molecular sexing [95], as well as to verify that they were free from any previous haemosporidian infections [96]. Twenty birds were experimentally inoculated on the 3^rd^ July 2010 (day 0, see Box 1 and Fig. 1) by means of an intraperitoneal injection of *ca.* 50–100 µL of an infected blood pool. The blood pool was constituted of a mixture of blood from 8 infected canaries that had been inoculated with the parasite 12 days previously following standard laboratory procedures [97]. Note that unlike what happens in some *Plasmodium* parasites such as *P. vivax*, the artificial infection with *P. relictum* via the inoculation of infected blood containing merozoites does not prevent the formation of exoerythrocytic stages [44], [98]. One bird failed to get infected and the remaining infected birds were assigned to two treatments: “exposed” (*n* = 10) or “unexposed” (control, *n* = 9) to mosquito bites. This assignment was made by balancing the gender of birds and the magnitude in the peak parasitaemia during the acute phase between the two treatments. This experimental design thus allowed mosquitoes to both probe and blood feed on the birds, and in this respect it contrasts with previous designs where only probing was allowed [39], [40].

Exposure to mosquito bites took place in August 2010 (first exposure session), and repeated in November 2010 and April 2011 (second and third exposure sessions). Each of these exposure sessions consisted of 3 “exposure days” separated by 3 day intervals: days 34, 37 and 40 post-infection (dpi) for the first exposure session, days 122, 125 and 128 dpi for the second exposure session, and days 291, 294 and 297 dpi for the third exposure session (Box 1 and Fig. 1A). In the morning of each exposure day, a small (*ca.* 15–25 µL) amount of blood was taken from the brachial vein of all (“exposed” and “control”) birds to quantify parasitaemia (see below). In the evening, birds allocated to the “exposed” treatment were placed inside a cage (dimensions L40×W30×H30 cm) with a batch of 50 uninfected female mosquitoes for 2 hours (8–10pm). Around 30 females blood fed on the birds during this time (see Table S3) which is close to available estimations in the wild [99]. Tables S2 and S3 provide the full details of the number of replications (number of blood fed mosquitoes, number of mosquitoes dissected) for each exposure session. To minimize host defensive behaviours that may alter the mosquito biting process during the assay, we immobilized birds in a specially designed PVC tube that rendered their legs accessible to the mosquitoes while protecting the rest of the body from the bites [97]. “Control” birds were placed in identical conditions but without mosquitoes. Immediately after each exposure, blood-fed mosquitoes from each cage (n = 10) and time point (3 exposure sessions, 3 days per session) were collected, isolated in a new cage, and maintained under standard laboratory conditions for 7 days. Fifteen haphazardly chosen mosquitoes were dissected to check for the presence (prevalence) and number (intensity) of oocysts in the midgut [94].

In each exposure session, two further blood samples were taken from all experimental birds, 3–4 days and 7–8 days after the last exposure day (days 44 and 48 dpi, days 131 and 135 dpi and days 300 and 304 dpi, for the first to third exposure sessions, Fig. 1B). For each exposure session we therefore obtained 5 different blood samples (red arrows in Fig. 1A and 1B). These blood samples were used to quantify total parasitaemia using previously published qPCR procedures [97] and gametocytaemia by microscopic examination (see Text S2). In addition, blood samples were taken at regular intervals throughout the experiment to monitor parasitaemia before and between the exposure sessions (blue arrows in Fig. 1A).

### Statistical analyses

The statistical analyses were run using the R software (v. 2.14.0). Analyses were carried out separately for each exposure session. Variation in parasitaemia (log-transformed (RQ+1)) was analyzed using linear mixed-effect models (*lme* function, *nlme* package) with bird as a random effect to account for the repeated sampling of individual hosts. A generalized linear mixed-effect models GLMM (*glmer* function, *lme4* package, binomial distribution) was carried out to study variation in gametocytaemia (proportion of gametocytes). Bird and time were included as random and fixed factors, respectively.

Variation in the infection prevalence (proportion of individuals harbouring at least 1 oocyst) and the oocystaemia (number of oocysts, only for infected mosquitoes) was analysed using GLMMs (*glmer* function, *lme4* package, with binomial and Poisson distributions, respectively). Bird and time (i.e. time between the 5 different blood samples, see red arrows in Fig. 1b) were included as random and fixed factors, respectively. Here, time was considered as a factorial explanatory variable.

When appropriate, *a posteriori* contrasts were carried out by aggregating factor levels that did not significantly differ from each other and by testing the fit of the simplified model [100]. The significance of explanatory variables was established by comparing the change in deviance with and without the term to a χ^2^ distribution. Degrees of freedom correspond to the difference in the number of terms in the model.

## Supporting Information

Table S1 Sample size for exposed and unexposed groups of birds across the experiment. Time points (days post-infection) refer to sampling times for the monitoring of blood parasitaemia.
(DOCX)

Table S2 Table summarizing the number of infected mosquitoes (over the 15 dissected) and oocyst burden (mean ± s.e.) for the 3 exposure sessions. Unexposed birds were kept as controls during the experiment, the birds that survived (see Table S1) were exposed once to mosquitoes at the end of the experiment (307 dpi).
(DOCX)

Table S3 Table summarizing the number of blood fed mosquitoes (around 50 were introduced into each cage) and, in parenthesis, the percentage of blood feeding success for the 3 exposure sessions. Unexposed birds were kept as controls during the experiment, the birds that survived (see Table S1) were exposed once to mosquitoes only at the end of the experiment (307 dpi).
(DOCX)

Text S1 Theory - Epidemiology and evolution of inducible transmission strategies.
(DOCX)

Text S2 Experiment - Quantification of gametocytaemia: Temporal variation and relationships with mosquito infection.
(DOCX)

Text S3 Experiment - Quantification of the effect of mosquito exposure on parasite transmission: A comparison of the differences in transmission between exposed and control birds.
(DOCX)
