# Supplementary material for: Evolution of Plastic Transmission Strategies in Avian Malaria
Source: PLoS Pathog. 2014 Sep 11;10(9):e1004308. doi: 10.1371/journal.ppat.1004308 (PMC4161439; doi:10.1371/journal.ppat.1004308)
Supplement: Table S1 — Sample size for exposed and unexposed groups of birds across the experiment. Time points (days post-infection) refer to sampling times for the monitoring of blood parasitaemia. (DOCX) [file ppat.1004308.s001.docx]

**Table S1:** Sample size for exposed and unexposed groups of birds across the experiment. Time points (days post-infection, dpi) refer to sampling times for the monitoring of blood parasitaemia.

| dpi | 0 | 9 | 14 | 20 | 30 | 34 | 37 | 40 | 44 | 48 | 73 | 104 | 122 | 125 | 128 | 131 | 135 | 165 | 195 | 227 | 250 | 291 | 294 | 297 | 300 | 304 |
| --- | --- | --- | --- | --- | --- | --- | --- | --- | --- | --- | --- | --- | --- | --- | --- | --- | --- | --- | --- | --- | --- | --- | --- | --- | --- | --- |
| Exposed | 10 | 10 | 10 | 10 | 10 | 10 | 10 | 10 | 10 | 10 | 10 | 10 | 9 | 8 | 7 | 7 | 7 | 7 | 7 | 6 | 6 | 6 | 6 | 5 | 4 | 4 |
| Unexposed | 9 | 9 | 9 | 9 | 9 | 9 | 9 | 9 | 9 | 9 | 9 | 9 | 9 | 9 | 9 | 9 | 9 | 9 | 9 | 8 | 8 | 6 | 6 | 5 | 3 | 3 |
