# Supplementary material for: Evolution of Plastic Transmission Strategies in Avian Malaria
Source: PLoS Pathog. 2014 Sep 11;10(9):e1004308. doi: 10.1371/journal.ppat.1004308 (PMC4161439; doi:10.1371/journal.ppat.1004308)
Supplement: Table S3 — Table summarizing the number of blood fed mosquitoes (around 50 were introduced into each cage) and, in parenthesis, the percentage of blood feeding success for the 3 exposure sessions. Unexposed birds were kept as controls during the experiment, the birds that survived (see Table S1) were exposed once to mosquitoes only at the end of the experiment (307 dpi). (DOCX) [file ppat.1004308.s003.docx]

**Table S3:** Table summarizing the number of blood fed mosquitoes (around 50 were introduced into each cage) and in parenthesis, the percentage of blood feeding success for the 3 exposure sessions. Unexposed birds were kept as controls during the experiment, the birds that survived (see Table S1) were exposed once to mosquitoes only at the end of the experiment (307 dpi).

|  |  | Session 1 |  |  | Session 2 |  |  | Session 3 |  |  |  |
| --- | --- | --- | --- | --- | --- | --- | --- | --- | --- | --- | --- |
|  |  | 34 | 37 | 40 | 122 | 125 | 128 | 291 | 294 | 297 | 307 |
| Exposed | Bird 1 | 30 (60.0) | 21 (42.0) | 23 (49.8) | 30 (60.0) | 34 (75.6) | 33 (66.0) | 21 (42.0) | 17 (34.7) | 19 (38.0) |  |
|  | Bird 2 | 21 (45.7) | 20 (40.0) | 24 (50.0) | 27 (54.0) | 35 (72.9) | 42 (84.0) | 24 (50.0) | 22 (44.0) | 14 (29.8) |  |
|  | Bird 3 | 24 (48.0) | 30 (61.2) | 29 (60.4) | 27 (52.9) | 37 (80.4) | 30 (58.8) | 15 (31.3) | 21 (42.0) | 22 (45.8) |  |
|  | Bird 4 | 19 (38.0) | 25 (50.0) | 31 (58.5) | 38 (76.0) | 33 (68.8) | 34 (69.4) | 21 (42.0) | 22 (44.0) | 16 (34.0) |  |
|  | Bird 5 | 24 (48.0) | 25 (50.0) | 23 (46.0) | 37 (75.5) | 34 (72.3) | 34 (64.2) | 20 (40.0) | 29 (59.2) | 19 (38.0) |  |
|  | Bird 6 | 25 (50.0) | 20 (43.5) | 25 (50.0) | 25 (51.0) | 33 (67.3) | 29 (60.4) | 14 (30.4) | 22 (45.8) |  |  |
|  | Bird 7 | 29 (60.4) | 31 (64.6) | 28 (57.1) | 34 (69.4) | 35 (74.5) | 38 (73.1) |  |  |  |  |
|  | Bird 8 | 25 (51.0) | 24 (49.0) | 23 (46.9) | 28 (58.3) | 36 (78.3) |  |  |  |  |  |
|  | Bird 9 | 21 (43.8) | 27 (54.0) | 24 (51.1) | 33 (66.0) |  |  |  |  |  |  |
|  | Bird 10 | 17 (34.7) | 28 (56.0) | 22 (44.0) |  |  |  |  |  |  |  |
| Unexp | Bird 11 |  |  |  |  |  |  |  |  |  | 16 (32.0) |
|  | Bird 12 |  |  |  |  |  |  |  |  |  | 16 (32.0) |
|  | Bird 13 |  |  |  |  |  |  |  |  |  | 35 (71.4) |
