# Supplementary material for: Evolution of Plastic Transmission Strategies in Avian Malaria
Source: PLoS Pathog. 2014 Sep 11;10(9):e1004308. doi: 10.1371/journal.ppat.1004308 (PMC4161439; doi:10.1371/journal.ppat.1004308)
Supplement: Text S1 — Theory - Epidemiology and evolution of inducible transmission strategies. (DOCX) [file ppat.1004308.s004.docx]

**TEXT S1**

**Theory – Epidemiology and evolution of inducible transmission strategies**

1. **Epidemiology**

Let us consider malaria epidemiology when vertebrate host population is assumed to be constant and equal to $N=S\left( t \right)+I\left( t \right)$, where $S\left( t \right)$ and $I\left( t \right)$ are the densities of uninfected and infected hosts, respectively. In contrast, the density of the vector population may change through time. This may be particularly relevant in temperate environments where the mosquitoes do not reproduce in winter. In other words, the influx $\theta\left( t \right)$ of uninfected mosquitoes is assumed to fluctuate periodically in time. As a consequence, the densities $V\left( t \right)$ and $V_{I}\left( t \right)$of uninfected and infected vectors also fluctuate through time. The following set of differential equation describes the temporal dynamics of the different types of hosts (the dot notation indicates differential over time):

| $S\left( t \right)=N-I\left( t \right)$  $\dot{I}\left( t \right)=S\left( t \right)V_{I}\left( t \right)\beta_{2}-\left( d+\alpha\right) I\left( t \right)$  $\dot{V}\left( t \right)=\theta\left( t \right)-I\left( t \right)V\left( t \right)\beta_{1}-m V\left( t \right)$  $\dot{V_{I}}\left( t \right)=I\left( t \right)V\left( t \right)\beta_{1}-m_{I}V_{I}\left( t \right)$ | (S1) |
| --- | --- |

where $d$ is the natural mortality rate of the vertebrate host and $\alpha$ is the virulence of malaria (the extra mortality induced by the infection); $m$ and $m_{I}$ are the mortality rates of uninfected and infected vectors, respectively; $\beta_{1}$ and $\beta_{2}$ are the transmission rates from the vertebrate host to the vector and vice versa, respectively.

The parameters $\alpha$ and $\beta_{1}$ may also vary with time when the pathogen is allowed to be plastic. The parameter $\varepsilon_{F}$ is the allocation towards a fixed level of virulence and $\varepsilon_{P}$ is the allocation to a plastic strategy that allows virulence to increase with the density of vectors. Transmission is assumed to be an increasing function of virulence and the shape of the relationship between virulence and transmission is governed by parameters $a$and $b$. In addition we assume that investing into a plastic trait may carry a direct fitness cost, $c$, and decrease transmission. This yields:

| $\alpha\left( t \right)=\varepsilon_{F}+\varepsilon_{P}\left( V\left( t \right)+V_{I}\left( t \right) \right)$  $\beta_{1}\left( t \right)=a \left[ \alpha\left( t \right) \right]^{b}-c \varepsilon_{P}$ | (S2) |
| --- | --- |

We can further simplify this model after assuming the turnover rates of the vector population is much faster than the one in the human host population. In this case the vector density will reach a quasi-equilibrium set by the temporal change in the influx of new vectors in the population and the prevalence of the disease in the human population at a given point in time:

| $V\left( t \right)=\frac{\theta\left( t \right)}{m-I\left( t \right)\beta_{1}}$  $V_{I}\left( t \right)=\frac{I\left( t \right)V\left( t \right)\beta_{1}}{m_{I}}$ | (S3) |
| --- | --- |

| Under this assumption the dynamics of the infection at the early stage of an epidemic (i.e. when $I\left( t \right)\sim0$) is given by:  $\dot{I}\left( t \right)=\underset{r_{0}\left( t \right)}{\underbrace{\left( N\frac{\theta\left( t \right)\beta_{1}\left( t \right)}{m m_{I}}\beta_{2}-\left( d+\alpha\left( t \right) \right) \right)}} I\left( t \right)$ | (S4) |
| --- | --- |

If we assume that the environment (i.e. $\theta\left( t \right)$) fluctuates with a period $T$ we integrate over one period of the fluctuation to obtain the necessary condition for the pathogen to spread:

| $\tilde{r_{0}}=\frac{1}{T}\int_{0}^{T} r_{0}\left( t \right)dt>0$ | (S5) |
| --- | --- |

In other words, a necessary condition for the epidemic to spread is:

| $\tilde{R}_{0}=\frac{N \beta_{2}}{\left( d+\tilde{\alpha} \right) m m_{I}}\left( \tilde{\theta}\tilde{\beta}_{1}+{COV}_{t}\left( \theta,\beta_{1} \right) \right)>1$ | (S6) |
| --- | --- |

where $\tilde{\theta}=\frac{1}{T}\int_{0}^{T} \theta\left( t \right)dt$, $\tilde{\beta}_{1}=\frac{1}{T}\int_{0}^{T} \beta_{1}\left( t \right)dt$, $\tilde{\alpha}=\frac{1}{T}\int_{0}^{T} \alpha\left( t \right)dt$ and ${COV}_{t}\left( \theta,\beta_{1} \right)$ is the covariance between $\theta\left( t \right)$ and $\beta_{1}\left( t \right)$.

Under the assumption that the amplitude of fluctuations of virulence remains small we can use the approximation:

| $\beta_{1}\left( t \right)\sim a \left[ \tilde{\alpha} \right]^{b}-c \varepsilon_{P}+ab \left[ \tilde{\alpha} \right]^{b-1}\left( \alpha\left( t \right)-\tilde{\alpha} \right)$ | (S7) |
| --- | --- |

Using (S2) and (S3) we have:

| $\alpha\left( t \right)=\varepsilon_{F}+\varepsilon_{P}\left( \frac{\theta\left( t \right)}{m} \right)$ | (S8) |
| --- | --- |

and consequently:

| ${COV}_{t}\left( \theta,\beta_{1} \right)=\varepsilon_{P}\frac{ab \left[ \tilde{\alpha} \right]^{b-1}}{m}{VAR}_{t}\left( \theta\right)$ | (S9) |
| --- | --- |

In other words, pathogen plasticity is expected to increase the viability of pathogen populations through its positive effect on the covariance between the increase in transmission and the availability of vectors.

1. **Evolution**

What are the consequences of this temporal variation of the availability of vectors on the evolution of malaria? To study this question we consider the fate of a mutant malaria strategy that would alter its life history strategy in the vertebrate host. In particular, we will consider that the traits $\varepsilon_{F}$ and $\varepsilon_{P}$ may vary among different genotypes. The dynamics of the density of malaria mutant $M$ is given by:

| $\dot{I}_{M}\left( t \right)=\left( \frac{S\left( t \right) \theta\left( t \right)\beta_{1M}\left( t \right)\beta_{2}}{m m_{I}}-\left( d+\alpha_{M}\left( t \right) \right) \right)I_{M}\left( t \right)$ | (S10) |
| --- | --- |

The dynamics of the frequency of malaria mutant $M$ is given by:

| $\dot{p}_{M}\left( t \right)=p_{M}\left( t \right)\left[ \left( \frac{S\left( t \right) \theta\left( t \right)\beta_{1M}\left( t \right)\beta_{2}}{mm_{I}}-\frac{S\left( t \right) \theta\left( t \right)\bar{\beta_{1}}\left( t \right)\beta_{2}}{m m_{I}} \right)-\left( \alpha_{M}\left( t \right)-\bar{\alpha}\left( t \right) \right) \right]$ | (S11) |
| --- | --- |

where the overbars refer to the average over the different malaria genotypes: $\bar{\alpha}\left( t \right)=\sum_{i} \left( p_{i}\left( t \right)\alpha_{i}\left( t \right) \right)$. If we assume that the environment (i.e. $\theta\left( t \right)$) fluctuates with a period $T$ we integrate over one period of the fluctuation, which yields:

| $s_{M}=\frac{1}{T}\int_{0}^{T} \left( {\dot{p}_{M}\left( t \right)}/{p_{M}\left( t \right)} \right)dt$  $=\frac{1}{T}\int_{0}^{T} \left( \left( \frac{S\left( t \right) \theta\left( t \right)\beta_{1M}\left( t \right)\beta_{2}}{m m_{I}}-\frac{S\left( t \right) \theta\left( t \right)\bar{\beta_{1}}\left( t \right)\beta_{2}}{m m_{I}} \right)-\left( \alpha_{M}\left( t \right)-\bar{\alpha}\left( t \right) \right) \right)dt$  $=\frac{\beta_{2}}{Tm m_{I}}\int_{0}^{T} \left( \left( S\theta\left( t \right)\left( \beta_{1M}\left( t \right)-\bar{\beta_{1}}\left( t \right) \right) \right) \right)dt-\left( \tilde{\alpha}_{M}-\tilde{\alpha} \right)$  $=\frac{\beta_{2}}{m m_{I}}\left( \tilde{S\theta}\left( \tilde{\beta}_{1M}-\tilde{\beta}_{1} \right)+{COV}_{t}\left( S\theta,\beta_{1M} \right)-{COV}_{t}\left( S\theta,\bar{\beta_{1}} \right) \right)-\left( \tilde{\alpha}_{M}-\tilde{\alpha} \right)$  $=\frac{\beta_{2}}{m m_{I}}\left( \tilde{S\theta} \Delta\beta_{1}+\Delta COV \right)-\Delta\alpha$ | (S12) |
| --- | --- |

where:

$S\theta\left( t \right)=S\left( t \right) \theta\left( t \right)$, $\tilde{S\theta}=\frac{1}{T}\int_{0}^{T} S\theta\left( t \right)dt$, $\tilde{\alpha}_{M}=\frac{1}{T}\int_{0}^{T} \alpha_{M}\left( t \right)dt$, $\tilde{\alpha}=\frac{1}{T}\int_{0}^{T} \alpha\left( t \right)dt$, $\tilde{\beta}_{1M}=\frac{1}{T}\int_{0}^{T} \beta_{1M}\left( t \right)dt$, $\tilde{\beta}_{1}=\frac{1}{T}\int_{0}^{T} \beta_{1}\left( t \right)dt$, $\Delta\beta_{1}=\frac{1}{T}\int_{0}^{T} \left( \beta_{1M}\left( t \right)-\beta_{1}\left( t \right) \right)dt$, $\Delta\alpha=\frac{1}{T}\int_{0}^{T} \left( \alpha_{M}\left( t \right)-\alpha\left( t \right) \right)dt$ and $\Delta COV= {COV}_{t}\left( S\theta,\beta_{1M} \right)-{COV}_{t}\left( S\theta,\beta_{1} \right)$.

A necessary condition for the spread of malaria mutant $M$is $s_{M}>0$ and the examination of equation S12 is very insightful regarding the evolution of time-dependent life history strategies. To better understand condition S12 one can rewrite the invasion condition in the following way (equation (4) in the main text):

| $s_{M}=\underset{\begin{aligned} Benefit of \\ transmission \end{aligned}}{\underbrace{\frac{\beta_{2}}{m m_{V_{I}}}\tilde{S\theta} \Delta\beta_{1}}}\underset{\begin{aligned} Cost of \\ virulence \end{aligned}}{\underbrace{-\Delta\alpha}}+\underset{\begin{aligned} Benefit of \\ plasticity \end{aligned}}{\underbrace{\frac{\beta_{2}}{m m_{V_{I}}}\Delta COV}}>0$ | (S13) |
| --- | --- |

The first term measures the benefit associated with the average effect of strategy $M$ on *transmission* over one period of the fluctuation (see also Kumo& Sasaki 2005). In particular, this term is positive if and only if $\left( \tilde{\beta}_{1M}-\tilde{\beta}_{1} \right)>0$. This benefit depends on the availability of susceptible hosts and this is why it also depends on $\frac{\beta_{2}}{m m_{V_{I}}}\tilde{S\theta}$.

The second term measures the cost associated with the average effect of strategy $M$ on *virulence* over one period of the fluctuation. This is the classical cost of virulence.

The third term measures the benefit associated with the covariation between *transmission* and the availability of susceptible hosts. This term indicates that there is selection on the match between the investment in transmission and the likelihood to find a susceptible host to infect. Both the density of the vertebrate and the vector hosts matter for transmission and this is why we recover the covariance between transmission with the product $S\theta\left( t \right)=S\left( t \right) \theta\left( t \right)$.

To illustrate the evolutionary dynamics resulting from the above model one may consider a scenario with additional assumptions regarding the possibility to change the functions $\beta_{1}\left( t \right)$and $\alpha\left( t \right)$. Let us assume that any plasticity function may evolve under the incompressible fluid constraint (see Gomulkiewicz& Kirkpatrick 1992) where averages over time remain constant. In other words, the allocation to transmission and virulence is assumed to be constant over a period of the fluctuation. In this case the first two terms in (S13) vanish and the only selective force acting on the pathogen population is the final term which tends to maximize the covariance between allocation to transmission and availability of susceptible hosts. In the case where the density of the vector population fluctuates over time, this selects for a higher investment in $\beta_{1}$ when vectors are around.

1. **Simulations**

We performed numerical simulations using *Mathematica 9.0* (Wolfram 2013) to study the epidemiology and the evolution of our model (code available upon request). We simulate the effect of seasonality using a periodic square wave for $\theta\left( t \right)$ (see equation (5) in the main text). Under the default parameter values used for our simulations there is a threshold value of the level of seasonality below which the malaria parasite goes extinct. In particular, in the absence of plasticity (S6) the default parameter values used below yield:

$$\tilde{R}_{0}=\frac{N \beta_{2} A a}{\left( d+a \right) m m_{I}}\left( 1-\tau\right)=\frac{1000}{202}\left( 1-\tau\right)$$

The critical value of seasonality below which the malaria population is driven to extinction is $\tau_{c}=0.798$. In the following we thus restrict our exploration to the cases where $\tau\in\left[ 0,0.75 \right]$.

First, we simulate a scenario in the absence of plasticity (i.e. $\varepsilon_{P}=0$) and we allow the malaria parasite to mutate on the trait governing, $\varepsilon_{F}$, the fixed exploitation strategy. We vary the seasonality of the environment and track the long term evolution of the investment in $\varepsilon_{F}$ (Fig. 3). Second we allow mutations to affect both the fixed, $\varepsilon_{F}$, and the plastic, $\varepsilon_{P}$, exploitation strategies. We study the impact of seasonality on the coevolution between these two traits (Fig. 4).

| **Parameters and variables** | **Definition** | **Default value** |
| --- | --- | --- |
| $N$ | Size of the vertebrate host population | 100 |
| $d$ | Natural mortality of the vertebrate host | 1 |
| $\alpha$ | Virulence of malaria on the vertebrate host | - |
| $\varepsilon_{F}$ | Fixed exploitation strategy | - |
| $\varepsilon_{P}$ | Plastic exploitation strategy | - |
| $m$ | Mortality rate of uninfected mosquitoes | 10 |
| $m_{I}$ | Mortality rate of infected mosquitoes | 10.1 |
| $\beta_{1}$ | Transmission rate form the vertebrate host to the mosquito | - |
| $a$ | Shape of the transmission virulence trade-off | 1 |
| $b$ | Shape of the trade-off | 0.5 |
| $c$ | Cost of plasticity | - |
| $\beta_{2}$ | Transmission rate form the mosquito to the vertebrate host | 1 |
| $\theta\left( t \right)$ | Periodic influx of uninfected mosquitoes | - |
| $T$ | Period of the fluctuations | 400 |
| $\tau$ | Seasonality (fraction of time unsuitable for vector) | - |
| $A$ | Maximal influx of uninfected mosquitoes | 10 |

The symbol “–“ indicates that we have used different parameter values or that the variable changes throughout the simulation.

**References**

Gomulkiewicz R, Kirkpatrick M (1992) Quantitative genetics and the evolution of reaction norms. Evolution 46: 390-411.

Kamo M, Sasaki A (2005) Evolution toward multi‐year periodicity in epidemics. EcolLett 8: 378-385.

Wolfram Research, Inc. (2013). Mathematica Edition: Version 9.0. Wolfram Research, Inc. Champaign, Illinois.
