# Supplementary material for: Evolution of Plastic Transmission Strategies in Avian Malaria
Source: PLoS Pathog. 2014 Sep 11;10(9):e1004308. doi: 10.1371/journal.ppat.1004308 (PMC4161439; doi:10.1371/journal.ppat.1004308)
Supplement: Text S2 — Experiment - Quantification of gametocytaemia: Temporal variation and relationships with mosquito infection. (DOCX) [file ppat.1004308.s005.docx]

**TEXT S2**

**Experiment - Quantification of gametocytaemia: Temporal variation and relationships with mosquito infection**

**Background**

Gametocytes are *Plasmodium* transmissible sexual parasite stages, infectious for the vectors. In sessions 2 and 3, we quantified gametocytaemia and investigated how it varied with exposure to mosquito bites and were related to mosquito infection.

**Methods**

There are currently no molecular methods available to specifically quantify gametocytes in avian malaria parasites. Gametocytaemia was therefore estimated by observing Giemsa-stained blood smears for 40 min (x 1000 magnification) and sorting parasites as asexual (trophozoites and merozoites) or sexual (gametocytes) according to their morphology as described in Valkiūnas (2005). Gametocytaemia was expressed as a proportion and calculated as the number of red blood cells with gametocyte stages over the number of total red blood cells infected by *Plasmodium* (asexual and sexual) parasites.

The statistical analyses were run using the R software (v. 2.14.0). Analyses were carried out separately for each exposure session. A generalized linear mixed-effect models GLMM (*glmer* function, *lme4* package, binomial distribution) was carried out to study variation in gametocytaemia (proportion of gametocytes). Bird and time were included as random and fixed factors, respectively.

**Results**

Gametocytaemia was only quantified in the second (122-135 dpi) and third (291-304 dpi) exposure sessions. In the second exposure session, birds exposed to mosquito bites had a significantly greater proportion of gametocytes than control birds (*time** *exposure*: χ^2^_1_ = 66.34, *P* = 0.0313, Fig. S2.1a), independently of the overall parasitaemia (χ^2^_1_ = 0.44, *P* = 0.5079). The reverse, however, seemed to be true during the third (291-304 dpi) exposure session (Fig. S2.1b), although here the exposure effect was not statistically significant; (*exposure*: χ^2^_1_ = 3.58, *P* = 0.0586, *time*: χ^2^_1_ = 4.48, *P* = 0.0342, *parasitaemia*: χ^2^_1_ = 0.81, *P* = 0.3687). Visual observation of the data, however, showed that the results for the non-exposed birds followed a bell-shaped curve. We therefore refitted the statistical models, this time using *time* as a factorial explanatory variable. These results confirm that on day 297, birds in the unexposed treatment had significantly more gametocytes than birds in the exposed treatment (χ^2^_1_ = 15.67, *P*< 0.0001).

We did not find that gametocytaemia affected mosquito infection rate (session 2 χ^2^_1_ = 2.39, *P* = 0.1221; session 3 χ^2^_1_ = 1.02, *P* = 0.3120) although this has to be taken with caution given the scarcity of data, i.e. low sample size, missing data or no parasite stage observed).

**References**

Valkiūnas G (2005) Avian Malaria Parasites and OtherHaemosporidia. Boca Raton, FL., USA: CRC Press.

**Figure S2.1** Temporal variation in gametocytaemia for the sessions of mosquito exposure in the final two exposure sessions: **(a)** session 2 (122-135 dpi) and **(b)** session 3 (291-304 dpi). Unexposed (open circles, dashed line) or exposed to mosquito bites (filled circles, solid line).

**
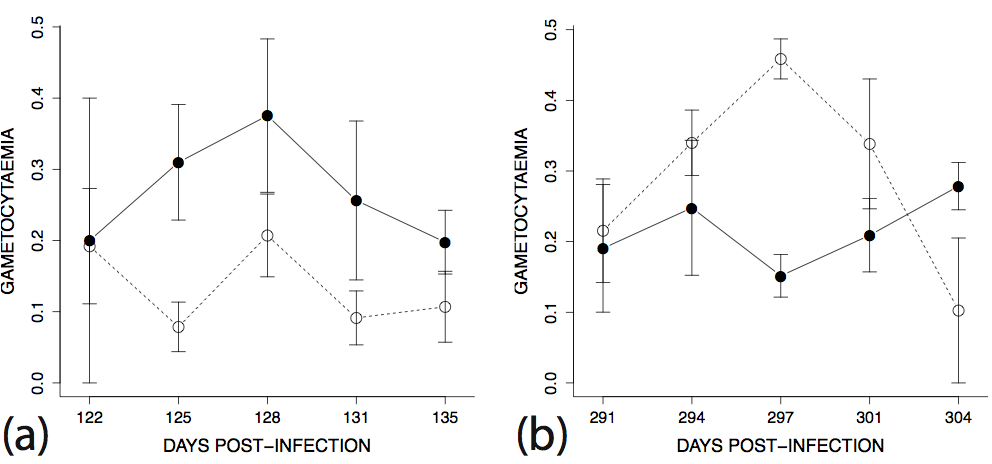
**
