# Supplementary material for: Evolution of Plastic Transmission Strategies in Avian Malaria
Source: PLoS Pathog. 2014 Sep 11;10(9):e1004308. doi: 10.1371/journal.ppat.1004308 (PMC4161439; doi:10.1371/journal.ppat.1004308)
Supplement: Text S3 — Experiment - Quantification of the effect of mosquito exposure on parasite transmission: A comparison of the differences in transmission between exposed and control birds. (DOCX) [file ppat.1004308.s006.docx]

**TEXT S3**

**Experiment - Quantification of the effect of mosquito exposure on parasite transmission: A comparison of the differences in transmission between exposed and control birds**

**Background**

In the main experiment (see main text) all the mosquitoes used in the same exposure session emerged roughly at the same date. As a consequence, females from the second and third mosquito batches were 3 and 6 days older (respectively) than mosquitoes used in the first batch. To control for a potential confounding effect of female age on transmission we therefore carried out another experiment using females of identical age (7 days old) at each exposure session.

**Methods**

The experiment was conducted in with a laboratory strain of *Culex pipiens* (SLAB). We used females 7 days after emergence that had had no prior access to blood, had been maintained on glucose solution (10%) provided with a cotton wick, and had been starved (i.e. provided with water only) for 6 h before the experiment. All rearing and experimental conditions were therefore identical to those of the main experiment (see materials and methods for details). Here, however, the 3 different batches of mosquitoes were all the same age: 7 days old.

We used domestic canaries harbouring a *Plasmodium relictum* (SGS1) chronic infection (54 to 171 days post parasite inoculation). Birds were haphazardly assigned either to the control “unexposed” (*n* = 7) or to the mosquito “exposed” (*n* = 7) treatment groups, making sure to homogenise genders, acute-stage parasitaemia and age of infection between groups.

The experiment was carried out in February 2012. Birds in the "exposed" group were exposed to mosquitoes every 3 days (same protocol as in the main experiment, Fig. 1b), henceforth termed days 0, 3 and 6. "Control" birds were only exposed once, on the third exposure day (day 6). The blood feeding rate of laboratory SLAB mosquitoes is greater than that of wild-caught mosquitoes (S. Cornet, pers. obs.). Therefore to standardize the biting rate across the two experiments, birds were exposed to a fewer number of SLAB females (n = 35), which were allowed to bite for 2h. On each "exposure day", but prior to the assay, a small amount (*ca.* 15-25 µL) of blood was taken from the brachial vein of each of the birds to measure parasitaemia (by qPCR) and host haematocrit (Cornet *et al.* 2013). We sampled blood for 2 more time-points (days 9 and 12) to follow the parasite's response after the last mosquito exposure. Blood fed mosquitoes were kept in the laboratory for 7 days and then dissected to check for the presence and number of oocysts in the midgut. Results were analysed according to the same statistical procedures as described in the main part of the manuscript.

**Results**

In agreement to our previous observations, the blood feeding success of SLAB mosquitoes was high (90.99 % ± 1.08) and constant across the 3 exposures (*F*_2,12_ = 1.33, P = 0.3020). As such, the number of biting SLAB mosquitoes in this experiment (31.67 ± 0.36) is almost similar to the biting pressure undergone by the birds in the main experiment using wild mosquitoes (26.20 ± 0.77). See Table S3.1

There was a slightly significant temporal variation in blood parasitaemia (χ^2^_1_ = 3.47, *P* = 0.0626) which was independent of whether the birds were exposed to the mosquitoes (exposure χ^2^_1_ = 0.0002, *P* = 0.9873, time*exposure χ^2^_1_ = 0.89, *P* = 0.3442) (Fig. S3.1a). Host haematocrit also remained unaffected by mosquito exposure (χ^2^_1_ = 0.007, *P* = 0.9349, Fig S3.1b), although exposed birds tended to have higher haematocrit following mosquito biting (likely resulting from the production of new erythrocytes).

Mosquito infection rate increased as birds were exposed to mosquito bites (χ^2^_2_ = 27.85, *P*< 0.0001, all contrasts being significant, Fig S3.2a). As shown before, mosquitoes of the second exposure time point had more oocysts (contrast (0+6) *vs.* 3 χ^2^_1_ = 10.04, *P* = 0.0015; Fig. S3.2b). Here, although mosquitoes had light infection, the results remained quantitatively similar compared to those obtained in the previous experiment (main text).

On day 6 (third biting session), both exposed and control birds were exposed to mosquitoes. This allowed us to establish whether previous exposure to bites increases malaria transmission. Mosquitoes tended to be more infected when biting birds previously exposed to bites than when they bit control (unexposed) birds (mean ± s.e. infection rates: 0.28 ± 0.05 and 0.21 ± 0.07 respectively, χ^2^_1_ = 2.90, *P* = 0.0885, Fig. S3.3a). There was, however, no difference in oocystaemia (mean ± s.e. oocysts, unexposed: 1.37 ± 0.11, exposed: 1.45 ± 0.10; χ^2^_1_ = 0.12, *P* = 0.7297, Fig. S3.3b). It is worth mentioning that, for unknown reasons, oocyst burdens were very low in this experiment.

**References**

Cornet S, Nicot A, Rivero A, Gandon S (2013) Malaria infection increases bird attractiveness to uninfected mosquitoes. EcolLett 16: 323-329.

**Table S3.1** Table summarizing the number of blood-fed SLAB mosquitoes and the percentage of blood feeding success for the 3 exposure sessions. Unexposed birds were kept as controls during the experiment, they were exposed only once to mosquitoes at day 6 post first exposure.

|  |  | Days post first exposure | | |
| --- | --- | --- | --- | --- |
|  |  | 0 | 3 | 6 |
| Exposed | Bird A | 28 (80.0%) | 31 (88.6) | 31 (88.6) |
|  | Bird B | 32 (94.1) | 33 (94.3) | 32 (91.4) |
|  | Bird C | 33 (94.3) | 31 (88.6) | 32 (91.4) |
|  | Bird D | 26 (74.3) | 31 (88.6) | 32 (91.4) |
|  | Bird E | 33 (97.1) | 31 (88.6) | 31 (88.6) |
|  | Bird F | 31 (93.9) | 30 (85.7) | 31 (88.6) |
|  | Bird G | 33 (97.1) | 30 (85.7) | 32 (91.4) |
| Unexp | Bird H |  |  | 35 (100.0) |
|  | Bird I |  |  | 35 (100.0) |
|  | Bird J |  |  | 33 (94.3) |
|  | Bird K |  |  | 30 (85.7) |
|  | Bird L |  |  | 32 (91.4) |
|  | Bird M |  |  | 34 (97.1) |
|  | Bird N |  |  | 34 (97.1) |

**Figure S3.1 (a)** Variation (mean ± s.e.) in blood parasitaemia (Log(RQ+1)), and **(b)** haematocrit in birds unexposed (open circles, dashed line) or exposed to mosquito bites (filled circles, solid line).

**
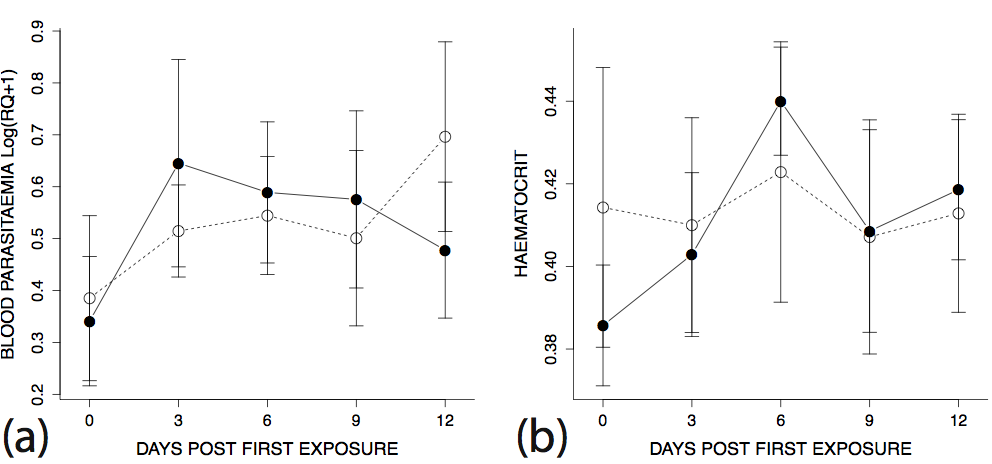
**

**Figure S3.2 (a)** Infection rate (proportion of infected mosquitoes, harbouring at least 1 oocyst in the midgut) and **(b)** oocystaemia (number of oocysts in mosquitoes harbouring ≥1 oocysts) for birds of the exposed group at the 3 different exposure dates.

**
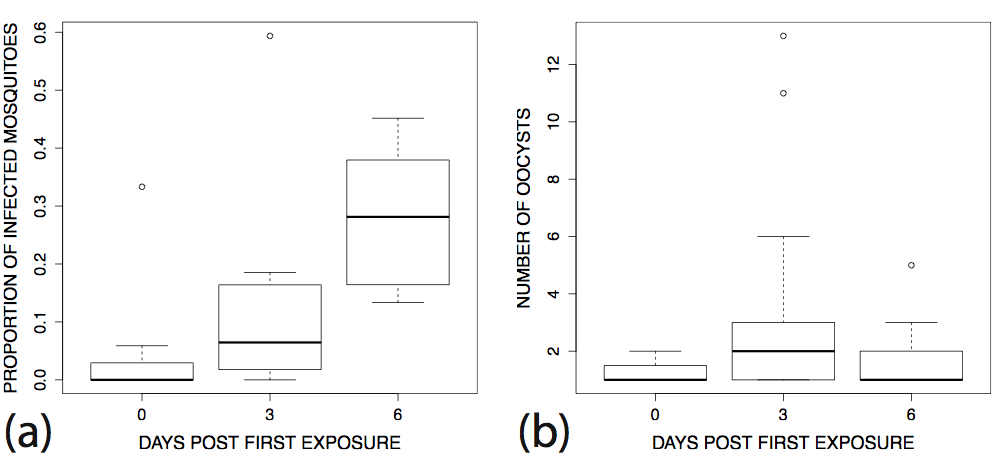
**

**Figure S3.3(a)** Infection rate and (proportion of infected mosquitoes, harbouring at least 1 oocyst in the midgut) and **(b)**oocystaemia (number of oocysts in mosquitoes harbouring ≥1 oocysts) of mosquitoes biting birds previously exposed to mosquito bites (“exposed” group) or left as control (“unexposed” group).

**
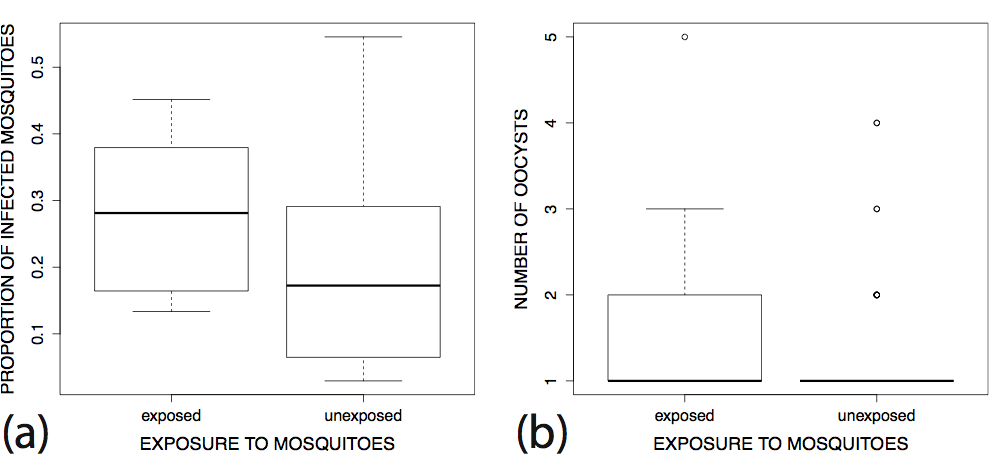
**
